# Sustained blood pressure reduction associated with percutaneous auricular vagus nerve stimulation in hypertensive chronic pain patients: a retrospective dual-center analysis

**DOI:** 10.3389/fcvm.2026.1736774

**Published:** 2026-03-30

**Authors:** Laurenz Berger, Rudolf Likar, Christophe Perruchoud, Stefan Kampusch, Van Hoang Le, Klaus Zeiner, Caroline Stremnitzer, Francesco Moscato, Max Haberbusch

**Affiliations:** 1 Center for Medical Physics and Biomedical Engineering, Medical University of Vienna, Vienna, Austria; 2 Ludwig Boltzmann Institute for Cardiovascular Research, Vienna, Austria; 3 Department for Anesthesia and Critical Care, Klinikum Klagenfurt am Wörthersee, Klagenfurt, Austria; 4 Sigmund Freud Privatuniversität Wien, Vienna, Austria; 5 Clinique de la Douleur, Hôpital de La Tour, Meyrin, Switzerland; 6 AURIMOD GmbH, Vienna, Austria; 7 Austrian Cluster for Tissue Regeneration, Vienna, Austria; 8 Department of Biomedical Engineering, George Washington University, Washington, DC, United States

**Keywords:** auricular vagus nerve stimulation, blood pressure regulation, chronic pain, hypertension, neuromodulation

## Abstract

**Background:**

Hypertension is a major risk factor for cardiovascular diseases, affecting over 1.28 billion adults worldwide, with nearly 46% remaining undiagnosed or untreated. Auricular vagus nerve stimulation (aVNS) has emerged as a promising non-invasive neuromodulation approach for autonomic regulation, yet its effects on blood pressure (BP) remain underexplored.

**Objective:**

The effects of aVNS on blood pressure in chronic pain patients were evaluated, with a specific focus on differential responses by hypertensive status and antihypertensive treatment.

**Methods:**

This retrospective dual-center study analyzed the impact of aVNS on BP in 24 chronic pain patients [mean age 48.5 (9.1) years; 75% female], categorized into non-hypertensive (*n* = 13) and hypertensive (*n* = 11) individuals. The hypertensive cohort was further stratified into patients receiving pharmacological hypertension treatment (*n* = 5) and those untreated (*n* = 6). Patients received aVNS over an 8-week treatment period, followed by a 4-week follow-up.

**Results:**

Over an 8-week treatment period, hypertensive patients exhibited significant reductions in systolic BP [−10.7 (2.9) mmHg, *p* = 0.0003] and diastolic BP [−5.8 (2.0) mmHg, *p* = 0.0357], while non-hypertensive individuals showed no significant BP changes. Subgroup analysis revealed that BP reductions were most robust and consistent in untreated hypertensive patients (SBP: −11.0 mmHg, *p* = 0.001), whereas patients on antihypertensive medication showed greater variability. Mean arterial pressure (MAP) declined significantly in hypertensive individuals [−9.3 (6.7) mmHg, *p* = 0.0022]. In contrast, no significant changes were observed in heart rate or heart rate variability [e.g., heart rate: −0.9 (1.8) beats/min; root mean square of successive differences in normal RR intervals: −12.8 (9.0) ms at week 12, both *p* = 1.0000], suggesting preserved autonomic stability.

**Conclusions:**

aVNS may be associated with BP reductions in hypertensive patients particularly those not receiving pharmacological treatment, with minimal effects in normotensive individuals. These retrospective findings suggest a potential sustained benefit in patients with comorbid chronic pain and support further investigation through prospective, sham-controlled trials to confirm efficacy and clarify underlying mechanisms.

## 1 Introduction

Cardiovascular diseases remain the leading cause of mortality worldwide, accounting for approximately 17.9 million deaths annually (1). Among modifiable risk factors, hypertension is one of the most prevalent, affecting over 1.28 billion adults globally, with a significant proportion remaining untreated or inadequately controlled (2). Resistant hypertension—defined as uncontrolled blood pressure (BP) despite the use of multiple antihypertensive medications – poses a major challenge (3). Even when pharmacological therapies are effective, adherence remains suboptimal due to side effects and the chronic nature of treatment, highlighting the need for novel non-pharmacological interventions to complement or replace conventional drug therapy (4).

In recent years, neuromodulation strategies such as baroreflex activation therapy and renal sympathetic denervation have been explored as adjunctive therapies for resistant hypertension (5, 6), but these approaches require invasive procedures, limiting their widespread adoption. Vagus nerve stimulation (VNS) has emerged as a promising alternative due to its well-established autonomic effects, with implantable cervical VNS already being utilized in epilepsy and depression treatment among others (7). However, surgical implantation remains a significant barrier for cardiovascular applications, prompting interest in non-invasive VNS techniques.

Consequently, many studies have demonstrated the benefits of auricular vagus nerve stimulation (aVNS) on autonomic modulation and BP reduction. As a non-invasive, easily accessible alternative, aVNS leverages vagal afferents in the external ear to modulate autonomic activity across various conditions, including depression (8), chronic pain (9), and heart failure (10–12). Recent randomized trials have expanded the evidence base, demonstrating that aVNS can effectively modulate autonomic balance in conditions such as postural orthostatic tachycardia syndrome (13). Furthermore, Jiang et al. (14) recently reported concurrent reductions in BP and heart rate in patients with paroxysmal atrial fibrillation, while observations in healthy volunteers suggest that hemodynamic effects may be minimal in the absence of autonomic pathology (15, 16). While its role in hypertension treatment remains underexplored, recent clinical trials suggest that aVNS can significantly lower BP in hypertensive individuals (17–20).

aVNS is thought to lower BP by enhancing baroreflex sensitivity and reducing sympathetic tone. Preclinical studies suggest that aVNS engages vagal afferents projecting to the nucleus tractus solitarius, modulating autonomic outflow (21). In humans, Antonino et al. (22) demonstrated that aVNS acutely increased baroreflex sensitivity by ∼24%, while Clancy et al. (23) showed a reduction in sympathetic tone, supporting a potential role in BP regulation. Recent advancements, such as respiratory-gated aVNS, have been shown to enhance autonomic engagement and optimize BP-lowering effects (24).

Despite promising findings, existing studies on aVNS for hypertension have varied in methodology and patient populations, contributing to mixed results (12). However, emerging randomized controlled trials suggest that long-term aVNS therapy can produce sustained BP reductions in hypertensive patients (20). In a recent randomized controlled trial, Mbikyo et al. (20) showed that daily aVNS sessions resulted in an average reduction of 8–14 mmHg in systolic BP over three months, providing strong evidence for its antihypertensive potential.

To further contribute to this growing body of evidence, we conducted a retrospective analysis of cardiovascular data from a clinical study of chronic pain patients treated with aVNS. Unlike previous studies that often focus on acute effects in controlled laboratory settings or exclude patients with significant comorbidities, this study evaluates the hemodynamic effects of long-term (12-week) aVNS therapy. Given that chronic pain has been associated with autonomic dysfunction and heightened sympathetic activity, making this “dual-burden” population a unique and clinically relevant cohort for assessing the robustness of aVNS. By analyzing the BP responses to aVNS in this real-world setting, our study aims to explore potential mechanisms and identify patient characteristics, and clinical applicability of this novel neuromodulatory approach. Given the retrospective nature of our analysis, we aimed to explore potential effects of aVNS on BP while acknowledging the inherent limitations in causal attribution.

## 2 Materials and methods

### 2.1 Study design and setting

This retrospective analysis was based on data collected from a multicenter study conducted between April 2021 and October 2022 at Klinikum Klagenfurt am Wörthersee, Austria and Hôpital de La Tour, Switzerland. The original study investigated the effects of aVNS on chronic low back pain. Ethical approvals for the original study were obtained from the respective local committees (ethics committee Carinthia M2020-30; ethics committee CCER Geneva 2020-01951) and was registered in EUDAMED (Ref.no.: CIV-20-09-034667). This analysis adhered to the Declaration of Helsinki.

### 2.2 Participants

**Figure 1:**
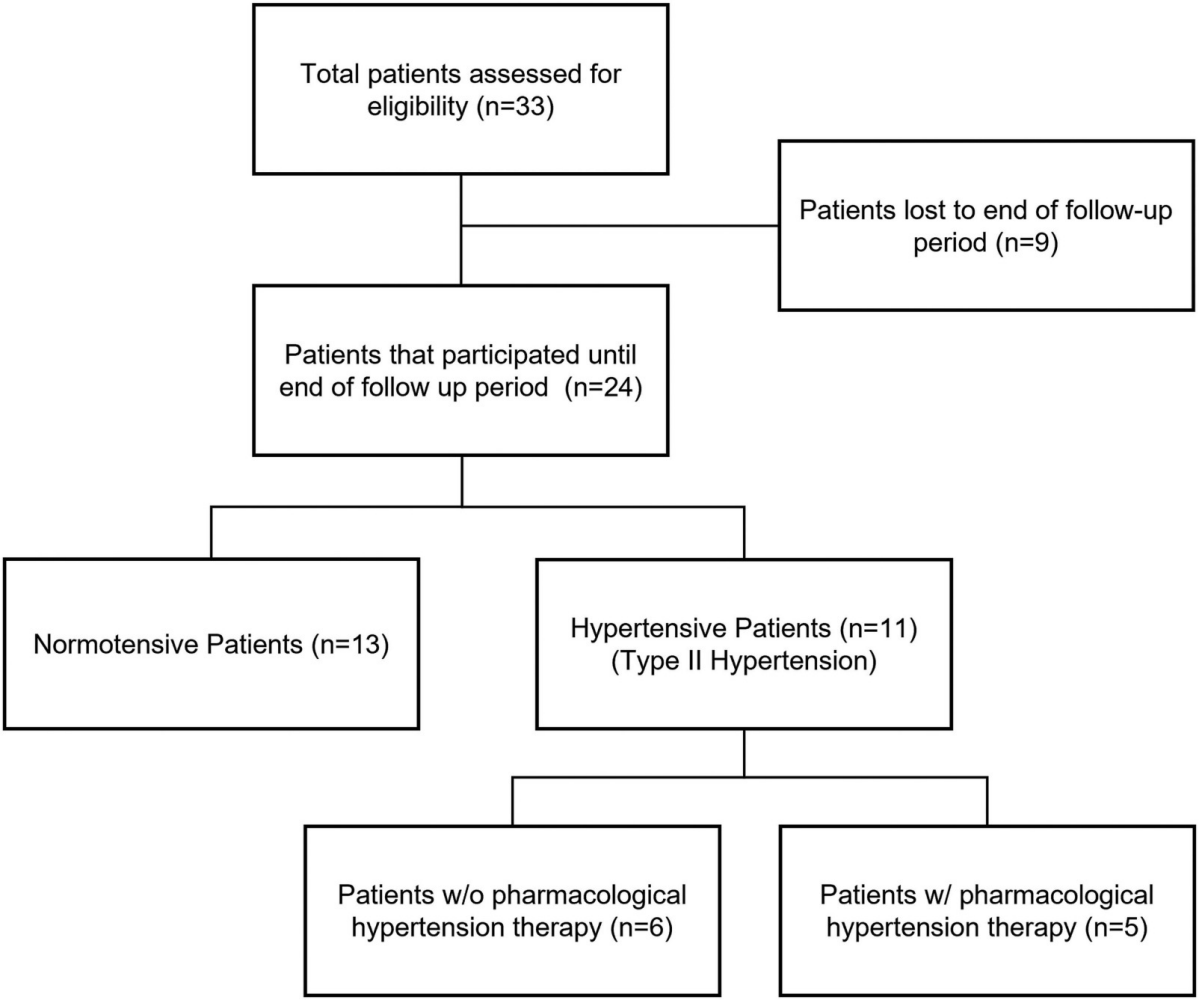
Flow diagram illustrating the patient selection process for analysis. A total of 33 patients were assessed for eligibility. Of these, 9 were lost to follow-up. The final analysis included 24 patients who completed all assessments. Patients were categorized into non-hypertensive (*n* = 13; SBP < 140 mmHg and DBP < 90 mmHg) and hypertensive (*n* = 11; SBP ≥ 140 mmHg or DBP ≥ 90 mmHg) groups. The hypertensive group was further subdivided into patients without pharmacological hypertension therapy (*n* = 6) and patients receiving pharmacological hypertension therapy (*n* = 5).

The analysis included 24 patients with chronic low back pain who were treated with aVNS. An overview of the patient selection process, including inclusion and exclusion criteria, is presented in Figure 1. Detailed eligibility criteria are provided in Supplementary Table S1. Medication regimen was stable prior to enrollment, and to avoid confounding autonomic effects, patients taking beta-blockers were excluded.

### 2.3 Intervention

Participants received chronic aVNS treatment using the AuriMod CT01 device (AURIMOD GmbH, Austria) delivering triphasic bursted stimulation at 1 Hz inter-burst frequency with rectangular pulses of 500 *μ*s pulse widths. Stimulation amplitude was adjustable between 0 and 5 V (constant voltage), to adapt to the individual perception level of patients. Duty cycle on/off periods varied between 120 and 180 min for the “on” phase and 20–120 min for the “off” phase. Details on the stimulation parameters can be found in earlier publications (25). The treatment was administered over a period of 8 weeks as part of a standard chronic pain management regimen. Each patient received aVNS continuously over a period of 7 days for 8 consecutive weeks. At each therapy visit (every week), a new device was applied. Patients were evaluated at baseline, weekly during treatment for 8 weeks, and during a 4-week follow-up.

**Figure 2:**
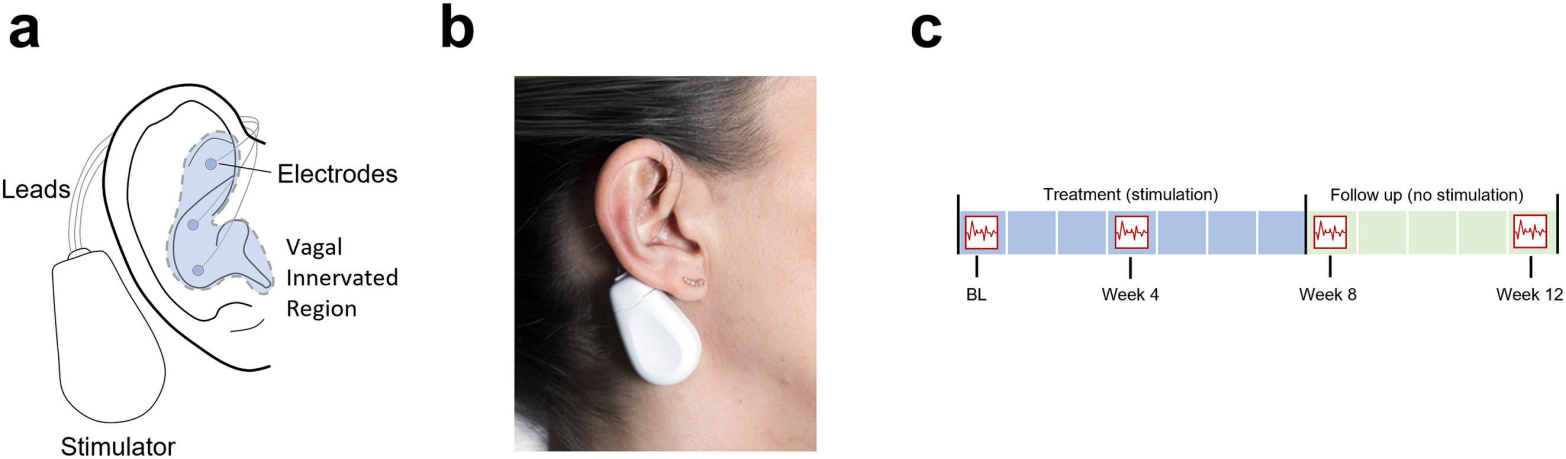
**(a)** Schematic representation of the tripolar electrode placement for auricular vagus nerve stimulation (aVNS). **(b)** Example of aVNS device placement on a patient's ear. **(c)** Timeline illustrating the intervention and outcome assessment periods, including baseline (BL, week 1), mid treatment (week 4), end of treatment (week 8), and end of follow-up (week 12).

Device placement was standardized across all participants, utilizing a tripolar electrode configuration targeting the auricular branch of the vagus nerve in the triangular fossa, cymba conchae, and cavity conchae region (Figures 2a,b). Percutaneous stimulation was delivered using single-shank, disposable titanium needle electrodes (27-gauge, 2 mm length). These electrodes were positioned in the above regions, partly or solely innervated by the auricular vagus nerve. The electrode positions were chosen close to local blood vessels running in parallel or near the targeted nerve fibers.

### 2.4 Data collection and variables

BP and heart rate (HR) were measured daily using a validated oscillometric device (Boso, Bosch + Sohn GmbH, Germany) during baseline (week 1), mid-treatment (week 4), end of treatment (week 8), and follow-up (week 12). For each period, mean BP and HR were calculated from all available daily measurements to enable comparison across time points. Nighttime heart rate variability (HRV) was calculated as the root mean square of successive differences (RMSSD) in normal RR intervals using data from the aVNS device, which continuously monitored HR and transmitted it via NFC to the smartphone app. At the end of follow-up (week 12), RMSSD was derived from a 24-hour Holter ECG, as the aVNS device was not used during the non-stimulation period. All data were anonymized before analysis to ensure patient confidentiality.

The primary outcome of this analysis was the change in systolic blood pressure (SBP), diastolic blood pressure (DBP), mean arterial pressure (MAP), HR, and RMSSD from base line to mid treatment (week 4), end of treatment (week 8), and end of follow-up (weeks 12). Stimulation was applied from baseline to end of week 8, while no stimulation was administered during the follow-up period (weeks 9–12). A timeline illustrating the different phases is presented in Figure 2c.

### 2.5 Statistical analysis

Patients were categorized as non-hypertensive or hypertensive based on SBP and DBP thresholds of 140 mmHg and 90 mmHg, respectively, consistent with the definition of stage-2 hypertension. Subgroup analyses were performed, distinguishing between (1) diagnosed hypertensive patients receiving hypertension medication, (2) untreated hypertensive patients, and (3) non-hypertensive individuals. Hypertensive classification was based on baseline BP measurements and remained fixed throughout the study for subgroup comparisons.

Data were statistically analyzed in Python using the SciPy and statsmodels packages. Normality of the data was assessed using the Shapiro–Wilk test. To account for the hierarchical structure of the data (i.e., repeated measures nested within individuals) and to accommodate occasional missing data points across visits, linear mixed models (LMMs) were employed. LMMs offer greater flexibility and robustness than repeated-measures ANOVA in handling unbalanced longitudinal data. Changes over time in SBP, DBP, MAP, HR and RMSSD were modeled using LMMs with time as a fixed effect and random intercepts for each subject to account for intra-individual variability. Estimated marginal means and their 95% confidence intervals were extracted from the model. Effect sizes were reported as estimated fixed-effect coefficients.

Pairwise comparisons between time points (baseline, week 4, week 8, and week 12) were conducted using *post hoc* testing on the LMM estimates. A total of six pairwise comparisons were performed, and Bonferroni correction was applied by adjusting *p*-values through division by the number of comparisons to control the family-wise error rate.

Additionally, fixed effects of stimulation voltage, body mass index and age, were explored in separate LMMs to assess potential covariate influence.

To contextualize the reliability of observed effects, *post hoc* power analysis was conducted for each patient group using the observed paired differences between baseline, treatment and follow-up time points. Cohen's d effect sizes and the corresponding statistical power (1 − *β*) were computed for all measured outcomes. This analysis allowed estimation of whether the observed within-group changes were detectable at conventional power thresholds, particularly given small sample sizes and a retrospective design.

To further explore individual variability in aVNS response, correlation analyses using Spearman's rank correlation coefficient were conducted to examine the relationship between baseline BP and HR with their respective reductions from baseline treatment and follow-up periods. Additionally, to assess the physiological concordance between analgesic and hemodynamic responses, Pearson correlation coefficients were calculated between the absolute change in visual analog scale (VAS) scores and the change in cardiovascular markers (SBP, DBP, MAP, HR, RMSSD). Responder status was visualized using clinical thresholds of ≥ 1.0 point reduction for VAS and ≥ 5.0 mmHg reduction for SBP.

Data were reported as mean (standard deviation) and 95% confidence intervals. For all statistical analyses, a *p*-value < 0.05 was considered statistically significant after correction.

## 3 Results

### 3.1 Characteristics

The cohort consisted of 24 patients, with a mean age of 48.5 (9.1) years, a mean body mass index of 28.4 (4.7) kg/m^2^, and 75.0% were female. Stimulation was delivered with a mean voltage of 525.3 (295.1) mV and mean on/off duty cycle periods of 121.7 (4.0) and 40.1 (2.5) min, respectively. Patients received medications including antihypertensives, analgesics (opioids and antipyretics), non-steroidal anti-inflammatory and anti-rheumatic drugs, antidepressants, muscle relaxants, and topical treatments for joint and muscular pain. Participants were grouped into non-hypertensive (*n* = 13) and hypertensive patients (*n* = 11). A subgroup analysis was conducted dividing the treated (*n* = 5) and untreated (*n* = 6) hypertensive patients. Table 1 summarizes the characteristics of the full cohort and the hypertensive and non-hypertensive subgroups.

**Table 1:** Characteristics of the entire patient population and the non-hypertensive and hypertensive subgroups.

Variable	All	Non-hypertensive	Hypertensive
Sex, number (%)			
Male	6 (25.0%)	3 (23.1%)	3 (27.3%)
Female	18 (75.0%)	10 (76.9%)	8 (72.7%)
Age (years), mean (SD)	48.5 (9.1)	48.4 (11.0)	48.7 (6.7)
95% CI	44.9 to 52.2	42.4 to 54.4	44.8 to 52.7
Body mass index (kg/m^2^), mean (SD)	28.4 (4.7)	27.6 (4.7)	29.4 (4.8)
95% CI	26.5 to 30.3	25.0 to 30.1	26.6 to 32.3
Height (cm), mean (SD)	170.2 (7.8)	168.5 (7.2)	172.2 (8.4)
95% CI	167.0 to 173.3	164.5 to 172.4	167.2 to 177.1
Weight (kg), mean (SD)	82.8 (17.2)	78.4 (15.3)	88.0 (18.7)
95% CI	75.9 to 89.7	70.1 to 86.7	77.0 to 88.0
Activity			
Steps/d (number), mean (SD)	5551 (3153)	6650 (3748)	4253 (1605)
95% CI	4290 to 6813	4613 to 8688	3304 to 5201
Stimulation Parameters			
Voltage (mV), mean (SD)	525.3 (295.1)	470.7 (119.4)	580.0 (405.1)
95% CI	53.1 to 1103.8	236.7 to 704.7	214.1 to 1374.0
Duty cycle on (min), mean (SD)	121.7 (4.0)	120.8 (2.4)	122.6 (5.1)
95% CI	113.9 to 129.5	115 to 125.6	112.7 to 132.6
Duty cycle off (min), mean (SD)	40.1 (2.5)	39.7 (0.8)	40.6 (3.5)
95% CI	35.2 to 45.1	38.1 to 41.3	33.6 to 47.5
Medication			
Hypertensive medication, number (%)	5 (20.8%)	0 (0%)	5 (45.5%)
Opioids, number (%)	12 (50.0%)	4 (30.8%)	8 (72.7%)
Other analgesics and antipyretics, number (%)	15 (62.5%)	9 (69.2%)	6 (54.6%)
Anti-inflammatory and anti- rheumatic products, number (%)	14 (58.3%)	9 (69.2%)	5 (45.5%)
Antidepressants, number (%)	1 (4.2%)	0 (%)	1 (9.1%)
Muscle relaxants, number (%)	1 (4.2%)	1 (7.7%)	0 (0.0%)
Topical products, number (%)	2 (8.3%)	2 (15.4%)	0 (0.0%)

### 3.2 BP and HR changes in hypertensive and non-hypertensive patients

**Figure 3:**
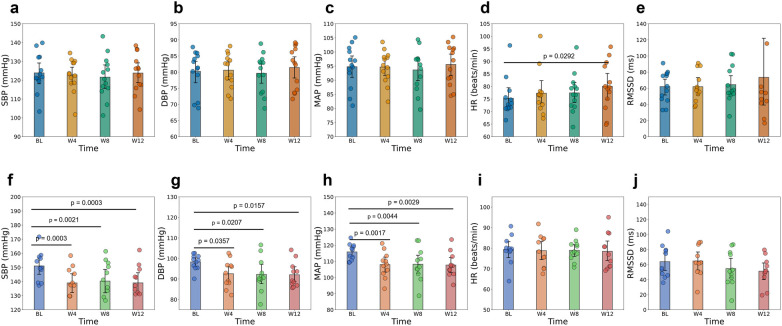
Mean and standard deviation in blood pressure, heart rate, and heart rate variability from baseline (BL, week 1) to mid-treatment (week 4), end of treatment (week 8), and end of follow-up (week 12) in non-hypertensive patients **(a–e)** and hypertensive patients **(f–j)**. Parameters include systolic blood pressure (SBP), diastolic blood pressure (DBP), mean arterial pressure (MAP), heart rate (HR), and root mean square of successive differences in normal RR intervals (RMSSD). Statistically significant pairwise comparisons (*p* < 0.05) are indicated above the corresponding bars. *Y*-axis limits are scaled independently for each panel to enhance visualization of within-group longitudinal changes.

**Figure 4:**
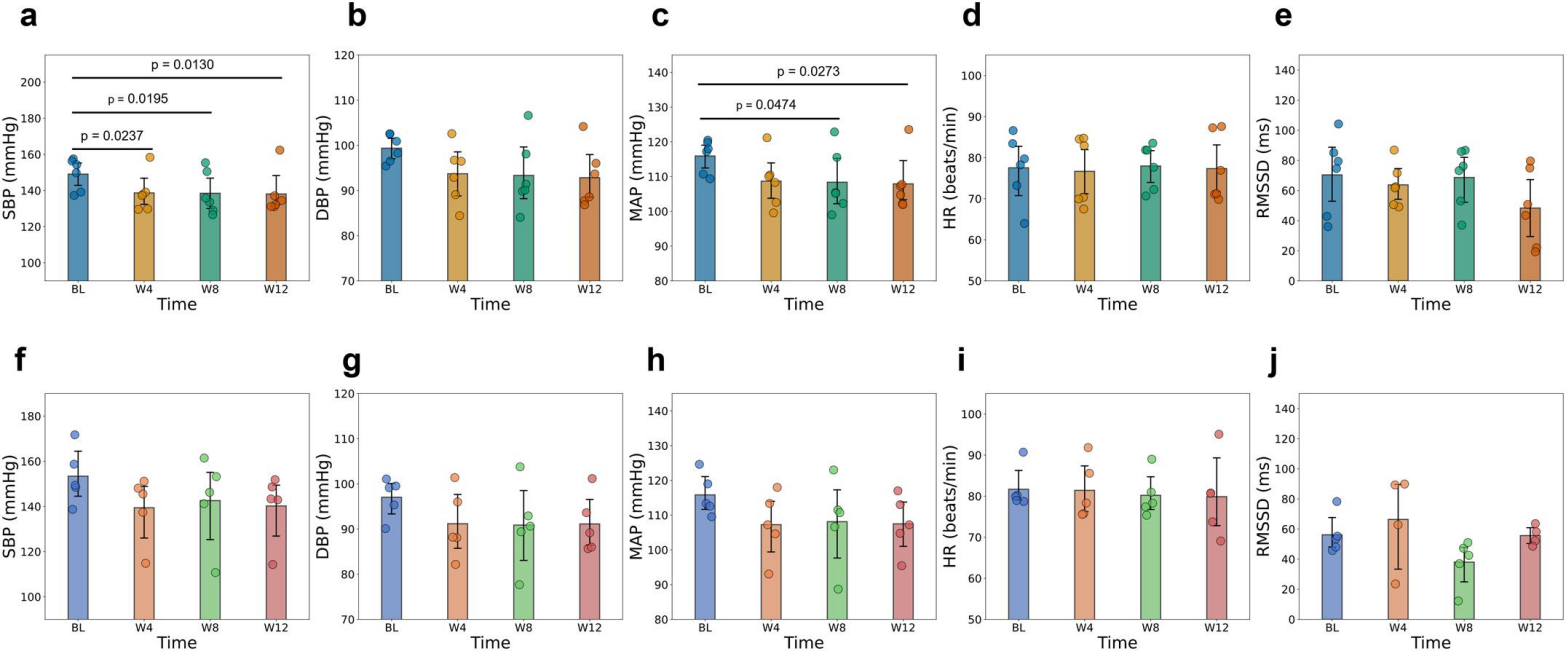
Mean and standard deviation in blood pressure, heart rate and heart rate variability markers from baseline (BL, week 1) to mid of treatment (week 4) to end of treatment (week 8) and end of follow up (weeks 12) in hypertensive patients without pharmacological hypertension treatment **(a–e)** and with pharmacological hypertension treatment **(f–j)**. Systolic blood pressure (SBP), diastolic blood pressure (DBP), mean arterial pressure (MAP), heart rate (HR), and root mean square successive difference in RR intervals (RMSSD). Statistically significant pairwise comparisons (*p* < 0.05) are indicated above the respective bars.

To assess the effects of aVNS over time, changes in BP, HR, and HRV were evaluated across the predefined study periods (Figure 3 and Supplementary Table S6). Additionally, to account for the confounding influence of medication, we stratified the hypertensive cohort into untreated (*n* = 6) and treated (*n* = 5) subgroups (Figure 4). Fixed effects of stimulation voltage, body mass index, and age were explored. These covariates showed negligible effects in the overall sample, though some subgroup-specific associations were identified (Supplementary Table S2).

#### 3.2.1 Group-level analysis of hypertensive and non-hypertensive patients

In hypertensive patients, aVNS significantly reduced SBP, DBP, and MAP over time (Figure 3 and Supplementary Table S6). SBP declined from 151.0 mmHg at baseline to 139.0 mmHg at week 4 (−12.0 mmHg, *p* = 0.0003), with reductions sustained through week 8 and week 12. DBP and MAP followed similar trajectories, with overall reductions of approximately 6 mmHg and 8 mmHg, respectively (all *p* < 0.05). In contrast, HR remained stable throughout the intervention and follow-up periods (*p* = 1.0000).

#### 3.2.2 Impact of antihypertensive medication

The subgroup analysis revealed distinct response patterns based on medication status. Untreated hypertensive patients exhibited robust and statistically significant reductions across all BP parameters (Figures 4a–c). From baseline to week 12, SBP decreased on average by 11.0 mmHg (*p* = 0.001), MAP by 8.0 mmHg (*p* = 0.003), and DBP by 6.5 mmHg (*p* = 0.011). These changes were highly consistent, as reflected by the narrow confidence intervals and large effect sizes (Supplementary Table S2). Furthermore, in this unconfounded group, stimulation voltage was significantly associated with SBP reduction (*p* < 0.001), indicating a clear dose-response relationship (Supplementary Table S5).

In contrast, treated hypertensive patients receiving agents such ACE inhibitors, showed comparable mean reductions in SBP (−13.2 mmHg at week 12) but failed to reach statistical significance across time points after correction (all *p* > 0.05). As illustrated in Figures 4f–h, this group displayed higher baseline variability and wider confidence intervals compared to the untreated group. Notably, HR and RMSSD remained statistically unchanged in both subgroups (Figures 4d,e,1,j), supporting the observation that BP reductions occurred without broad alterations in cardiac autonomic tone.

Non-hypertensive patients showed no significant changes in SBP, DBP, or MAP across time points (all *p* = 1.0000). HR displayed a modest upward trend, increasing from 75.3 (7.6) beats/min at baseline to 80.1 (9.6) beats/min at week 12 (+4.8 beats/min, *p* = 0.0292, Cohen's d = 0.6), though the clinical relevance remained uncertain. RMSSD remained statistically unchanged [62.0 (19.2) ms at baseline compared to 73.5 (76.5) ms at week 12; *p* = 0.410, d = 0.24], indicating stable autonomic tone throughout the study.

These results suggest that aVNS selectively lowers BP in hypertensive individuals without inducing significant changes in HR or HRV. However, the null findings for HR and HRV—especially in the non-hypertensive group—may reflect limited statistical power (d < 0.3, power < 15%) and should therefore be interpreted with caution. Individual trajectories and intersubject variability are shown in Supplementary Figure S1, S2, and full estimates are presented in Supplementary Tables S2, S3. RMSSD remained statistically unchanged [62.0 (19.2) ms at baseline compared to 73.5 (76.5) ms at week 12; *p* = 0.410, d = 0.24], indicating stable autonomic tone throughout the study (Supplementary Table S2).

#### 3.2.3 Correlation analysis of baseline blood pressure and heart rate with aVNS response

**Figure 5:**
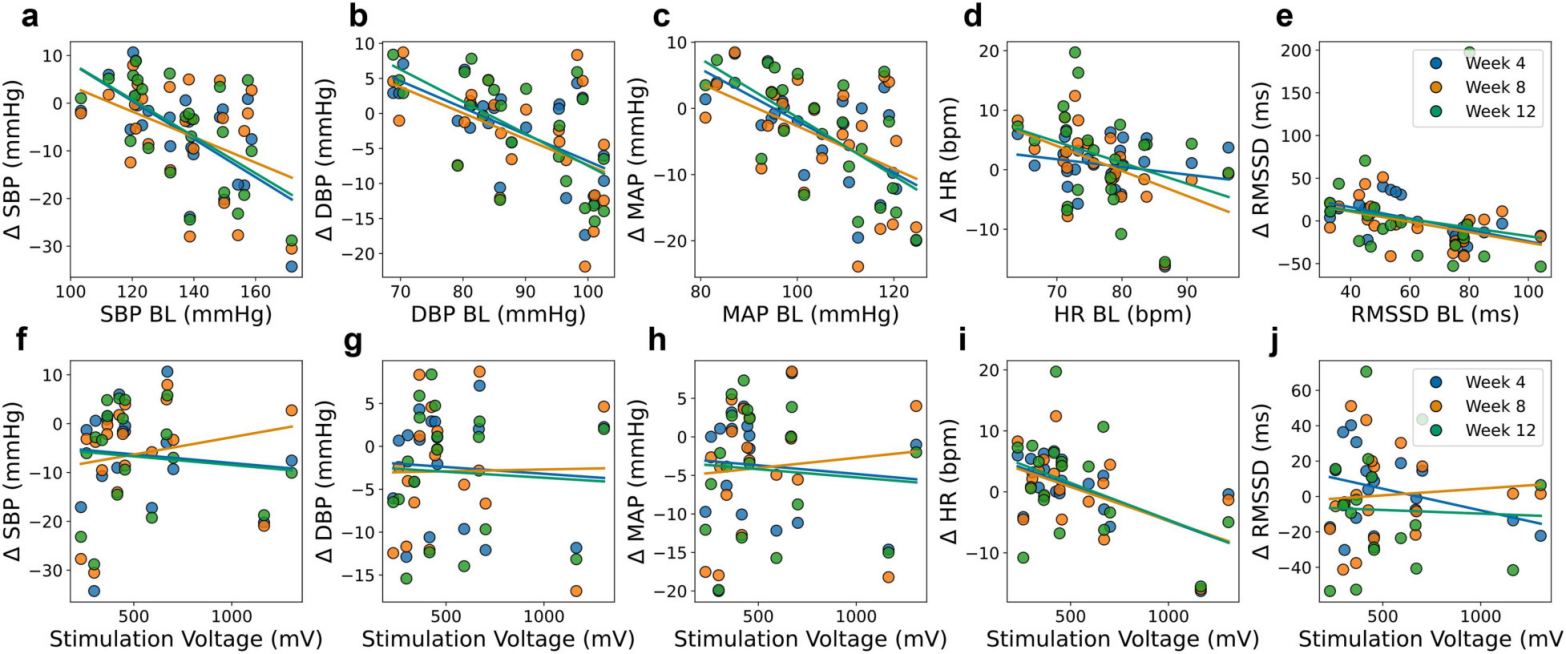
Relationship between cardiovascular markers at baseline value (week 1) and absolute change at mid-treatment (week 4), end of treatment (week 8), and end of follow-up (week 12) in the entire patient population **(a–e)** and relationship between absolute changes from baseline as a function of stimulation voltage **(f–j)**. Parameters include systolic blood pressure (SBP), diastolic blood pressure (DBP), mean arterial pressure (MAP), heart rate (HR), and root mean square of successive differences in normal RR intervals (RMSSD).

As depicted in Figure 5, Spearman correlation analysis revealed significant negative correlations between baseline BP values and their subsequent reductions, with stronger effects observed at end of follow-up (see Supplementary Table S4 for full statistical details).

Baseline SBP was significantly correlated with its reduction at mid-treatment (week 4, r = −0.59, *p* = 0.002) and end of follow-up (week 12, r = −0.57, *p* = 0.004), while the correlation at end of treatment (week 8: r = −0.33, *p* = 0.113) did not reach statistical significance. Similar trends were observed for DBP, which showed significant negative correlations at all time points (week 4: r = −0.60, *p* = 0.002; week 8: r = −0.50, *p* = 0.012; week 12: r = −0.65, *p* = 0.001). MAP reductions were also significantly correlated with baseline MAP (week 4: r = −0.61, *p* = 0.001; week 8: r = −0.40, *p* = 0.053; week 12: r = −0.64, *p* = 0.001), indicating that individuals with higher initial BP experienced greater reductions following aVNS.

In contrast, baseline HR did not show a consistent correlation with BP reductions, with significant associations observed only at end of treatment (week 8: r = −0.63, *p* = 0.001), while correlations at mid-treatment (week 4: r = −0.16, *p* = 0.458) and end of follow-up (week 12: r = −0.40, *p* = 0.053) were not statistically significant. Interestingly, RMSSD at baseline was significantly correlated with its reduction, particularly at end of follow-up (week 12: r = −0.71, *p* = 0.001), suggesting that patients with higher initial HRV values exhibited more pronounced decreases over time.

Stimulation voltage was neither significantly correlated with BP reductions at any time point (SBP: week 4, r = −0.03, *p* = 0.900; week 8, r = 0.44, *p* = 0.066; week 12, r = 0.11, *p* = 0.663), nor did it show meaningful associations with DBP or MAP changes (Supplementary Table S5). However, voltage demonstrated a significant negative correlation with HR reduction at mid-treatment (week 4: r = −0.54, *p* = 0.021), suggesting that higher stimulation intensities may be linked to greater HR decreases. No significant correlations were observed between stimulation voltage and RMSSD changes across time points.

#### 3.2.4 Concordance between analgesic and hemodynamic responses

**Figure 6:**
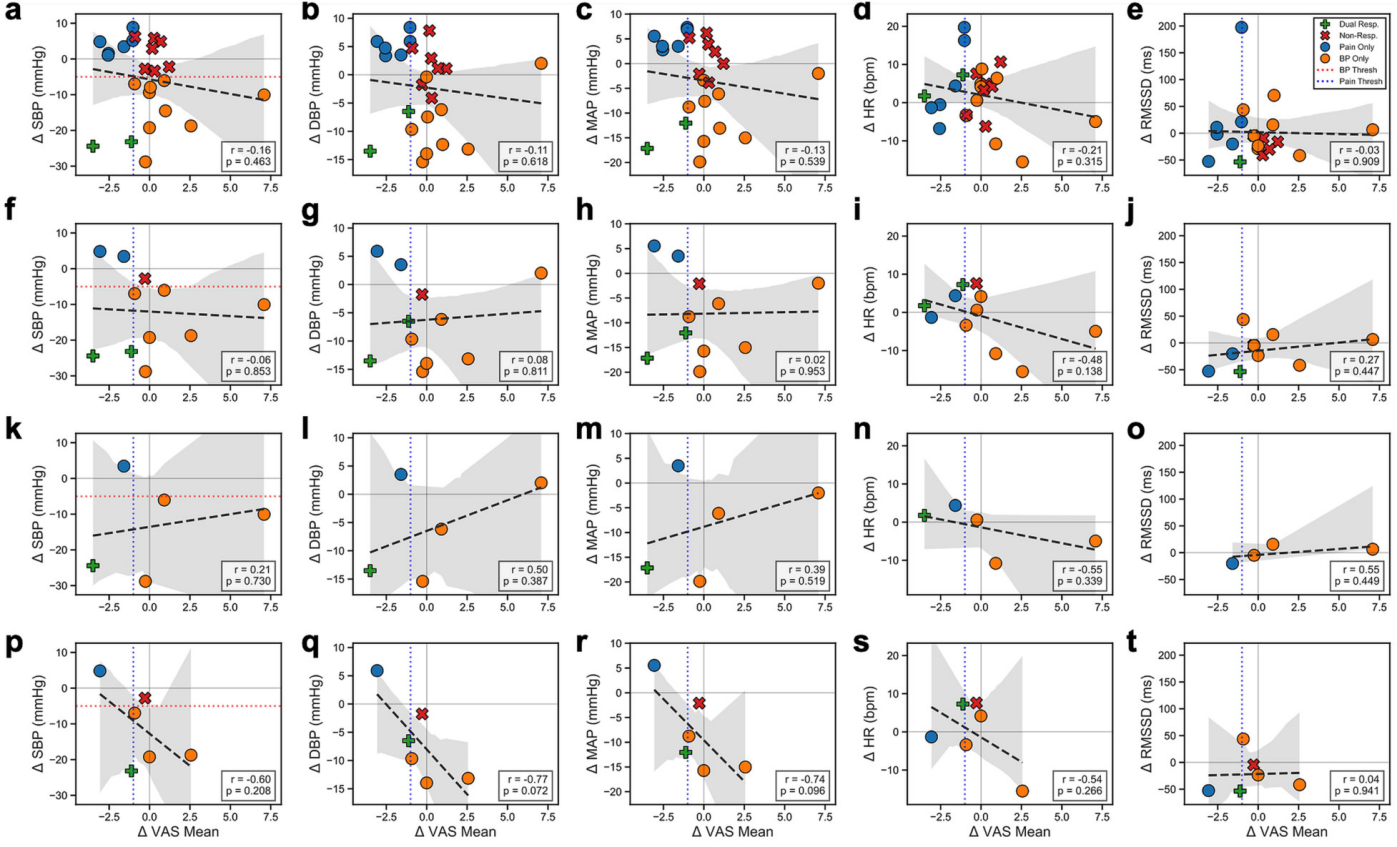
Concordance analysis of hemodynamic vs. analgesic responses across patient subgroups. Scatter plots illustrating the relationship between the change in mean visual analog scale (VAS) score and changes in systolic blood pressure (SBP), diastolic blood pressure (DBP), mean arterial blood pressure (MAP), heart rate (HR), and root mean square of successive differences (RMSSD) in normal RR intervals. Rows represent patient subgroups: **(a–e)** All Patients, **(f–j)** Total Hypertensive, **(k–o)** Hypertensive with Medication, and **(p–t)** Hypertensive without Medication. Note the strong negative correlation in untreated hypertensive patients (panel **p**), indicating that greater pain relief is associated with greater SBP reduction. This relationship is absent in medicated patients (panel **k**) suggesting a decoupling of the analgesic-autonomic response. Green crosses indicate “Dual Responders” (responders for both Pain and BP). Dotted lines represent clinical responder thresholds (VAS: −1.0; SBP: −5.0 mmHg).

To determine if patients who responded to pain treatment also responded to hypertension treatment, we analyzed the correlation between changes in VAS and cardiovascular markers (Figure 6). In the overall population, analgesic and hemodynamic responses appeared independent (r = −0.16, *p* = 0.463 for SBP). Subgroup analysis confirmed this trend, particularly in untreated hypertensive patients, where we observed a moderate negative correlation (SBP: r = −0.60, *p* = 0.208; DBP: r = −0.77, *p* = 0.072). This directionality indicates that large reductions in BP often occurred in patients who did not experience concurrent pain relief. In contrast, medicated patients showed a weak, non-significant positive trend (r = 0.21, *p* = 0.730), further suggesting that analgesic and cardiovascular improvements are distinct clinical outcomes rather than strictly coupled phenomena in this cohort.

## 4 Discussion

To our knowledge, this is the first study to demonstrate significant, sustained BP reductions specifically in hypertensive patients suffering from chronic pain—a population often excluded from standard hypertension trials due to the confounding effects of pain-related sympathetic arousal and analgesic medication. These findings extend previous literature by showing that the antihypertensive benefits of aVNS are not limited to controlled laboratory settings or pure essential hypertension but are translatable to complex, real-world comorbid populations over a 12-week treatment period.

The findings suggest that aVNS may contribute to reductions in BP in hypertensive individuals with limited observable effects in normotensive participants. These observations are in general agreement with a growing body of literature examining the autonomic and cardiovascular effects of aVNS. Although meta-analyses have indicated limited BP-lowering effects overall (12), recent studies have reported more pronounced outcomes in specific patient populations. For instance, a 2024 randomized controlled trial described SBP reductions of 8–14 mmHg following three months of daily tragus stimulation in hypertensive patients (20). Similarly, Jiang et al. (14) recently reported concurrent reductions in BP and HR in patients with paroxysmal atrial fibrillation, while Stavrakis et al. (10) demonstrated that aVNS reduced inflammatory markers and improved diastolic function in patients with heart failure with preserved ejection fraction. Such findings may indicate that longer-term stimulation could yield cumulative effects not apparent in earlier acute trials. Crucially, the observation that BP reductions in our study persisted through the 4-week follow-up period (Week 12) supports this notion of a sustained effect, suggesting lasting autonomic remodeling beyond the active stimulation window.

Garcia et al. (18) reported acute, frequency-dependent BP reductions—most pronounced at 100 Hz—with minimal HR changes following respiratory-gated aVNS in hypertensive adults. Similarly, we observed BP decreases in the hypertensive subgroup without significant changes in HR or HRV. While these findings may point toward baroreflex involvement, the absence of direct physiological measures (e.g., baroreflex sensitivity, sympathetic tone) in our study limits mechanistic interpretation. This caution is echoed by Stavrakis et al. (13), who found that aVNS attenuated autonomic instability in patients with postural orthostatic tachycardia syndrome, possibly through baroreflex modulation.

The minimal BP changes observed in normotensive individuals align with previous studies. For example, Šinkovec et al. (16) and Yoshida et al. (15) both reported no significant BP or HRV changes after low-level tragus stimulation in healthy adults, though some sex-specific cardiac responses were noted. These findings suggest that baseline cardiovascular status may influence aVNS responsiveness. Crucially, the absence of BP changes in the normotensive cohort serves as an internal reference, suggesting that the reductions observed in hypertensive patients are likely driven by physiological mechanisms dependent on baseline autonomic state rather than non-specific placebo effects.

This potential dependence on baseline state is echoed in the work by Maestri et al. (11), who reported increased HRV in healthy subjects but minimal changes in heart failure patients following left tragus stimulation at 25 Hz. In the present study, aVNS was associated with BP reductions in hypertensive participants but did not appear to significantly alter HR or HRV, possibly implying a distinct mechanism of action. Previous studies suggest that aVNS may modulate baroreflex sensitivity without markedly altering autonomic tone (22), a framework consistent with our findings. BP reduction may depend on baseline levels, while modest effects on HR and HRV could involve distinct or additional mechanisms. Stimulation intensity may influence HR, though its role in BP regulation remains unclear.

While exploratory subgroup analyses indicated that stimulation voltage and untreated hypertensive status may be associated with greater BP reductions, these effects were not statistically robust across all models and are detailed in the Supplementary Table S5. Age and body mass index were also found to be significant in certain subgroup models. While these associations are exploratory and should be interpreted with caution, they may point to individual factors that modulate responsiveness to aVNS and merit further investigation in controlled studies.

Stimulation site may also influence outcomes. Percin et al. (19) found that in-ear stimulation elicited more favorable BP and HRV changes than stimulation behind the ear, suggesting the importance of precise vagal targeting. Although we did not systematically compare stimulation sites, the BP effects observed in hypertensive patients suggest that the chosen parameters were sufficient to engage relevant neural pathways.

Taken together, these findings may support the notion that aVNS could exert BP-lowering effects in selected populations, particularly untreated hypertensive individuals and those with sympathetic-driven comorbidities like chronic pain, with minimal autonomic disturbance in those without elevated BP.

### 4.1 Hypothesized mechanism of action

**Figure 7:**
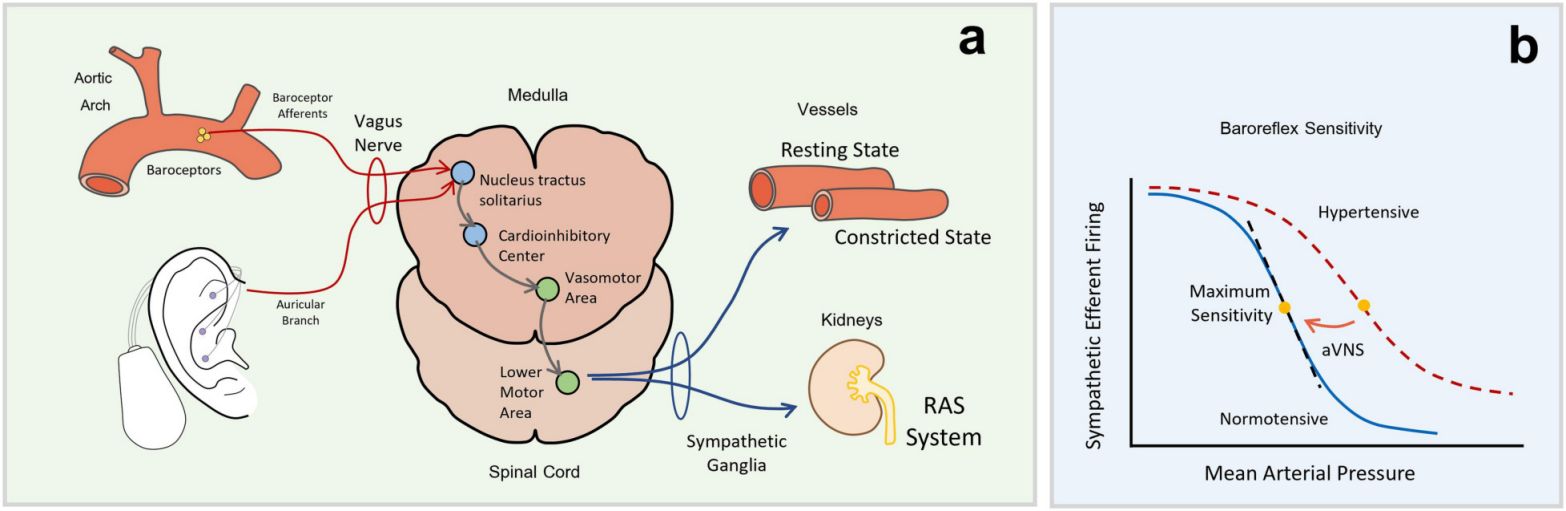
Proposed mechanism of action. **(a)** Schematic representation of the baroreflex pathway, highlighting the role of baroreceptors in the aortic arch, the vagus nerve, and the medulla in regulating sympathetic output to control vascular tone and kidney function. Baroreceptor afferents transmit signals to the nucleus tractus solitarius (NTS) in the medulla, which influences the cardiac control centers, including the vasomotor area, lower motor area, and the cardioinhibitory center, leading to adjustments in blood vessel constriction and renal function via the sympathetic ganglia and the renin-angiotensin system (RAS). **(b)** Baroreflex sensitivity curve, showing the relationship between sympathetic efferent firing and mean arterial pressure. The solid blue line represents normal baroreflex function, with maximum sensitivity occurring at a specific pressure range. The red dashed line illustrates impaired baroreflex sensitivity in hypertensive conditions. Auricular vagus nerve stimulation (aVNS) is hypothesized to influence baroreflex sensitivity, although this mechanism was not directly assessed in the present study. This schematic is conceptual and intended for illustrative purposes only. Mechanistic pathways were not empirically tested in this study.

The mechanisms through which aVNS may influence BP remain incompletely understood. One proposed explanation involves enhancement of baroreflex function, potentially without inducing broad shifts in autonomic tone. Stimulation of auricular vagal afferents may activate the nucleus tractus solitarius, a brainstem region involved in baroreceptor processing (Figure 7a), which could modulate cardiovascular control (Figure 7b).

Our concordance analysis (Figure 6) suggests that while aVNS can modulate both pain and BP, these effects may be distinct rather than obligatorily coupled. In the untreated hypertensive subgroup, we observed a negative correlation (r = −0.60, *p* = 0.208), driven largely by patients who experienced significant blood pressure reductions despite minimal analgesic response. This suggests that aVNS may successfully engage baroreflex circuits even when nociceptive modulation is insufficient, or that distinct “responder phenotypes” exist. Clinically, this is encouraging, as it implies that the antihypertensive benefits of aVNS are not contingent upon successful pain relief, allowing for cardiovascular therapeutic utility even in refractory pain patients.

Antonino et al. (22) observed that aVNS acutely increased baroreflex sensitivity by approximately 24% in healthy males, although no significant BP change was reported. These results might suggest that baroreflex enhancement can occur independently of acute BP effects, aligning with the present observations. Similarly, Clancy et al. (23) found that aVNS showed a reduction sympathetic tone, raising the possibility that reduced peripheral resistance may contribute to BP regulation.

Napadow et al. (24) investigated respiratory-gated aVNS, reporting that timing stimulation with the respiratory cycle amplified autonomic effects, including BP and HR modulation. Although respiratory-gating was not employed in the current study, such findings may help explain the variability in BP response across different protocols and highlight the importance of stimulation timing and parameter optimization.

In addition, sympathetic inhibition via aVNS might influence renal physiology by reducing renal sympathetic nerve activity and downstream renin–angiotensin system activation. Excess renal sympathetic tone is known to promote renin release and sodium retention, contributing to elevated angiotensin II levels and vasoconstriction (26). Attenuation of this pathway has been associated with BP reductions in renal denervation studies (26–29). While speculative, aVNS could potentially exert similar modulatory effects, although further physiological evidence is required to support this hypothesis.

The absence of HR and HRV changes in our study might suggest that aVNS, when effective, selectively modulates baroreflex circuits without triggering compensatory cardiac responses. This could reflect a targeted autonomic modulation rather than generalized vagal activation.

### 4.2 Clinical implications

Although preliminary, the current findings contribute to the evolving discussion around non-pharmacological interventions for hypertension. Traditional device-based approaches such as baroreflex activation therapy and renal denervation have demonstrated efficacy in certain populations, but their invasiveness and associated risks limit widespread application. Baroreflex activation therapy, for example, has been associated with SBP reductions of ∼24 mmHg (30), but requires surgical implantation with non-negligible complication rates. Renal denervation has shown more modest BP effects (∼6.6 mmHg office, ∼4.4 mmHg ambulatory) (27), with variable patient response.

In contrast, aVNS is non-invasive, portable, and relatively low-cost, potentially making it suitable for patients who are either intolerant to medications or prefer non-drug options. While the BP reductions observed here and in other studies (20) may not match those of invasive therapies, the risk-benefit profile of aVNS could make it attractive for certain patient groups, particularly in outpatient or home-based settings where managing comorbidities such as chronic pain alongside hypertension is a priority. Unlike daily self-administered transcutaneous systems, the percutaneous approach used here requires weekly clinical visits for device replacement. However, this model ensures consistent electrode placement and hygiene while allowing for continuous, unsupervised therapeutic delivery in the home environment between visits.

### 4.3 Limitations and future directions

This retrospective study was not prospectively powered for autonomic endpoints and lacked a dedicated sham or blank control group, limiting causal inference. BP reductions observed in hypertensives may partly reflect regression to the mean, as patients with elevated baseline values often show spontaneous decreases on repeat measurement. Nevertheless, the consistency of changes across visits and their association with stimulation parameters suggest that regression alone is unlikely to account for the findings, underscoring the need for confirmation in controlled trials. Indeed, *post hoc* power analysis (Supplementary Table S3) confirms that the study was sufficiently powered to detect the large effect sizes observed for BP reductions in hypertensive patients, where SBP reductions achieved Cohen's d values ≥ 1.04 and statistical power > 93%. In the untreated hypertensive subgroup, SBP reductions reached Cohen's d values between 0.97 and 1.11 with power estimates exceeding 65%. Furthermore, the identification of a significant dose-response relationship between stimulation voltage and SBP reductions (*β* = −0.02 mmHg/mV, *p* < 0.001) in this subgroup (Supplementary Section S1) supports a robust physiological effect rather than random variation. While sufficient power may have existed (generally < 15%, Supplementary Table S3) to detect larger BP changes in hypertensives, HR and HRV-related outcomes and effects in normotensives remain underpowered. The absence of significant HR or HRV changes should therefore be interpreted cautiously.

Interpretation is further limited by potential confounding from medications, pain levels, and visit frequency. While inclusion criteria required stable medication regimen, specific interactions between aVNS and these pharmacological agents could not be analyzed due to the small sample size. Although adjustments were made for age, BMI, and stimulation intensity, unmeasured variables may have influenced the results.

Physiological endpoints such as frequency-domain HRV parameters (e.g., LF/HF ratio), baroreflex sensitivity, sympathetic nerve activity, and catecholamine levels were not assessed, restricting mechanistic insight. Furthermore, as blood pressure was assessed via spot measurements rather than 24-hour ambulatory blood pressure monitoring, we were unable to evaluate circadian patterns, such as nighttime dipping status, or granular daily kinetic effects. Due to the retrospective nature of the study and the technical specifications of the monitoring devices, analysis was restricted to time-domain HRV (RMSSD). As such, while some observations are suggestive of baroreflex involvement, definitive conclusions cannot be drawn without direct physiological assessment.

Exploratory regression models suggested possible associations between stimulation voltage and BP reductions, and indicated roles for individual factors such as age and BMI. However, these findings may reflect sampling variability or uncontrolled confounders and require prospective validation.

Future work should prioritize sham-controlled, prospective trials with standardized stimulation protocols, autonomic phenotyping, and longitudinal follow-up. Such studies are needed to clarify the therapeutic potential of aVNS in hypertension and determine its place among existing interventions.

## 5 Conclusion

Our analysis suggests that aVNS may be associated with BP reductions in hypertensive patients—particularly those not on antihypertensive medication—while having minimal effects in normotensive individuals. These findings are among the first to suggest that such benefits may extend to chronic pain populations, a group traditionally excluded from hypertension trials despite high comorbidity rates. These findings align with emerging evidence supporting aVNS as a non-invasive neuromodulatory approach for cardiovascular modulation. Notably, BP reductions appeared to persist beyond the stimulation period, suggesting a potential sustained benefit. While these retrospective results require confirmation in prospective, sham-controlled trials, aVNS could offer a promising non-pharmacological option for patients with hypertension, particularly those with medication intolerance or those in complex chronic pain populations, where standard antihypertensive titration can be challenging.

## Data Availability

The original contributions presented in the study are included in the article/supplementary material, further inquiries can be directed to the corresponding author/s.
