# Correction: Sustained blood pressure reduction associated with percutaneous auricular vagus nerve stimulation in hypertensive chronic pain patients: a retrospective dual-center analysis

**DOI:** 10.3389/fcvm.2026.1856537

**Published:** 2026-05-06

**Authors:** Laurenz Berger, Rudolf Likar, Christophe Perruchoud, Stefan Kampusch, Van Hoang Le, Klaus Zeiner, Caroline Stremnitzer, Francesco Moscato, Max Haberbusch

**Affiliations:** 1 Center for Medical Physics and Biomedical Engineering, Medical University of Vienna, Vienna, Austria; 2 Ludwig Boltzmann Institute for Cardiovascular Research, Vienna, Austria; 3 Department for Anesthesia and Critical Care, Klinikum Klagenfurt am Wörthersee, Klagenfurt, Austria; 4 Sigmund Freud Privatuniversität Wien, Vienna, Austria; 5 Clinique de la Douleur, Hôpital de La Tour, Meyrin, Switzerland; 6 AURIMOD GmbH, Vienna, Austria; 7 Austrian Cluster for Tissue Regeneration, Vienna, Austria; 8 Department of Biomedical Engineering, George Washington University, Washington, DC, United States

**Keywords:** auricular vagus nerve stimulation, blood pressure regulation, chronic pain, hypertension, neuromodulation

The acknowledgements were missing. The correct acknowledgements are: “The authors thank Dr. Constantin Szeles and Dr. Eugenijus Kaniusas for their contributions to the original clinical study.”

The original version of this article has been updated.

