# Supplementary material for: Sustained blood pressure reduction associated with percutaneous auricular vagus nerve stimulation in hypertensive chronic pain patients: a retrospective dual-center analysis
Source: Front Cardiovasc Med. 2026 Mar 30;13:1736774. doi: 10.3389/fcvm.2026.1736774 (PMC13070907; doi:10.3389/fcvm.2026.1736774)
Supplement: Supplementary file 1 [file Datasheet1.pdf]

Sustained blood pressure reduction associated with percutaneous auricular vagus nerve stimulation in hypertensive chronic pain patients: A retrospective dual-center analysis

Supplementary material

**Inclusion criteria:**	• Male or female aged ≥ 18 and ≤ 65 years at screening<br>• Patient has an understanding of the study and its procedures, agrees to its provisions, and gives written informed consent prior to any study-related procedures<br>• Chronic back pain with or without leg pain (CBLP) persisting for at least 3 months<br>• A minimum of 70 (out of 180) points on the Computer User Self-Efficacy Scale at the screening visit<br>• Patient is constant with respect to pain treatment during the screening phase<br>• A daily average visual analog scale score (VAS) ≥ 4 on at least half of the days in the screening phase<br>• Compliance with the daily status reporting requirements as demonstrated by having valid required data entries for all days. It is acceptable if data of one day is missing in the screening phase
**Exclusion criteria:**	• Patients with age under 18<br>• Hemophilia<br>• Infection, eczema, or psoriasis at application site<br>• Numbed and desensitized skin at the application site<br>• Florid malignant diseases<br>• Mental and physical impairments that represent a source of risk for handling the device<br>• The presence of a cardiac pacemaker, defibrillator, cochlear implant, or other active implantable device<br>• Vagal hypersensitivity<br>• Indication for back surgery<br>• High-grade spinal stenosis<br>• Patients with other active implants<br>• Patients with autonomic disorders<br>• Patients with diabetes type I or II<br>• Patients taking Beta-Blockers<br>• Patients taking drugs carrying the potential risk of arrhythmia (tricyclic medications, Alzheimer drugs, etc.)<br>• Clinically significant hip or knee arthritis<br>• Allergy against rescue medication used during the study<br>• History of Vagus Nerve Stimulation<br>• Pregnant or nursing female patients<br>• Patients with arrhythmia, bradycardia, other rhythm disorders or any other clinically significant cardiac anomalies detected during ECG at screening

Supplementary Table 1 Overview of inclusion and exclusion criteria.

	**Effect size**	**95% CI**	**z**	**p-value**[a]	**p-value corrected**[b]
		All			
SBP (mmHg), Coeff. (SE)					
Week 4 vs BL	-6.1 (1.8)	-9.6 to -2.6	-3.418	0.001	**0.0069**
Week 8 vs BL	-6.1 (1.8)	-9.6 to -2.7	-3.450	0.001	**0.0062**
Week 12 vs BL	-5.5 (1.8)	-9.0 to -2.1	-3.121	0.002	**0.0199**
Stimulation voltage	+0.0 (0.0)	-0.01 to +0.02	0.808	0.419	1.0000
Body mass index	+0.4 (0.6)	-0.87 to +1.65	0.602	0.547	1.0000
Age	+0.2 (0.3)	-0.36 to +0.71	0.650	0.516	1.0000
DBP (mmHg), Coeff. (SE)					

Week 4 vs BL	-2.4 (1.2)	-4.8 to -0.1	0.284	0.046	0.5045
Week 8 vs BL	-3.1 (1.2)	-5.5 to -0.7	-0.458	0.011	0.1191
Week 12 vs BL	-2.2 (1.2)	-4.6 to 0.2	0.929	0.071	0.7782
Stimulation voltage	-0.0 (0.0)	-0.01 to +0.01	-0.008	0.994	1.0000
Body mass index	+0.2 (0.3)	-0.43 to +0.89	0.686	0.493	1.0000
Age	+0.1 (0.1)	-0.20 to +0.36	0.615	0.538	1.0000
MAP (mmHg), Coeff. (SE)					
Week 4 vs BL	-3.7 (1.3)	-6.3 to -1.0	-2.721	0.006	0.0715
Week 8 vs BL	-4.1 (1.3)	-6.7 to -1.5	-3.071	0.002	**0.0235**
Week 12 vs BL	-3.3 (1.3)	-6.0 to -0.7	-2.475	0.013	0.1465
Stimulation voltage	+0.0 (0.0)	-0.01 to +0.01	0.421	0.674	1.0000
Body mass index	+0.3 (0.4)	-0.42 to +1.08	0.694	0.488	1.0000
Age	+0.1 (0.2)	-0.22 to +0.45	0.680	0.496	1.0000
HR (beats/min), Coeff. (SE)					
Week 4 vs BL	+0.8 (1.2)	-1.5 to +3.2	0.691	0.490	1.0000
Week 8 vs BL	+1.0 (1.2)	-1.4 to + 3.4	0.810	0.418	1.0000
Week 12 vs BL	+2.2 (1.2)	-0.2 to + 4.6	1.787	0.074	0.8131
Stimulation voltage	+0.0 (0.0)	-0.01 to +0.01	0.252	0.801	1.0000
Body mass index	-0.2 (0.4)	-1.09 to +0.61	-0.551	0.582	1.0000
Age	-0.1 (0.2)	-0.43 to +0.29	-0.397	0.691	1.0000
RMSSD (ms), Coeff. (SE)					
Week 4 vs BL	+2.5 (8.4)	-14.0 to +19.0	0.297	0.767	1.0000
Week 8 vs BL	-2.9 (8.2)	-18.9 to +13.1	-0.354	0.723	1.0000
Week 12 vs BL	+0.1 (8.7)	-16.9 to +17.1	0.008	0.994	1.0000
Stimulation voltage	-0.0 (0.0)	-0.04 to +0.02	-0.486	0.627	1.0000
Body mass index	+1.4 (1.4)	-1.34 to +4.10	0.997	0.319	1.0000
Age	-0.5 (0.6)	-1.67 to +0.65	0.393	0.393	1.0000
Non-hypertensive					
SBP (mmHg), Coeff. (SE)					
Week 4 vs BL	-1.0 (1.7)	-4.4 to +2.4	-0.590	0.555	1.0000
Week 8 vs BL	-1.3 (1.7)	-5.7 to +1.2	-1.294	0.196	1.0000
Week 12 vs BL	-0.1 (1.7)	-3.5 to +3.3	-0.044	0.965	1.0000
Stimulation voltage	+0.0 (0.0)	-0.03 to +0.05	0.375	0.708	1.0000
Body mass index	+1.1 (0.9)	-0.6 to +2.8	0.129	0.195	1.0000
Age	-0.0 (0.4)	-0.8 to +0.8	-0.007	0.995	1.0000
DBP (mmHg), Coeff. (SE)					
Week 4 vs BL	+0.4 (1.3)	-2.2 to +2.9	0.284	0.777	1.0000
Week 8 vs BL	-0.6 (1.3)	-3.2 to +2.0	-0.458	0.647	1.0000
Week 12 vs BL	+1.2 (1.3)	-1.3 to +3.8	-0.929	0.353	1.0000
MAP (mmHg), Coeff. (SE)					
Week 4 vs BL	-0.1 (1.4)	-2.8 to +2.6	-0.071	0.944	1.0000
Week 8 vs BL	-1.2 (1.4)	-3.8 to +1.5	-0.849	0.396	1.0000
Week 12 vs BL	+0.8 (1.4)	-1.9 to +3.4	0.578	0.563	1.0000
Stimulation voltage	+0.0 (0.0)	-0.02 to +0.03	0.251	0.802	1.0000
Body mass index	+0.8 (0.6)	-0.24 to +1.94	1.524	0.128	1.0000
Age	+0.0 (0.3)	-0.49 to +0.50	0.030	0.976	1.0000
HR (beats/min), Coeff. (SE)					
Week 4 vs BL	+2.1 (1.6)	-1.2 to +5.3	1.258	0.208	1.0000
Week 8 vs BL	+2.2 (1.6)	-1.0 to +5.4	1.338	0.181	1.0000
Week 12 vs BL	+4.8 (1.6)	+1.6 to +8.0	2.943	0.003	**0.0292**
Stimulation voltage	-0.0 (0.0)	-0.04 to +0.03	-0.057	0.954	1.0000
Body mass index	-0.3 (0.8)	-1.25 to +1.90	0.403	0.687	1.0000
Age	-0.3 (0.4)	-1.02 to +0.40	-0.849	0.397	1.0000
RMSSD (ms), Coeff. (SE)					

Week 4 vs BL	+4.5 (13.6)	-22.2 to +31.3	0.331	0.740	1.0000
Week 8 vs BL	+2.4 (13.3)	-23.5 to +28.4	0.183	0.855	1.0000
Week 12 vs BL	+11.9 (14.5)	-16.4 to +40.3	0.825	0.410	1.0000
Stimulation voltage	-0.0 (0.1)	-0.14 to +0.12	-0.142	0.887	1.0000
Body mass index	+1.7 (2.9)	-4.03 to +7.50	0.590	0.555	1.0000
Age	-0.7 (1.3)	-3.27 to +2.00	-0.486	0.627	1.0000
Hypertensive					
SBP (mmHg), Coeff. (SE)					
Week 4 vs BL	-12.0 (2.9)	-17.7 to -6.4	-4.167	<0.001	**0.0003**
Week 8 vs BL	-10.7 (2.9)	-16.4 to +5.1	-3.708	<0.001	**0.0021**
Week 12 vs BL	-12.0 (2.9)	-17.7 to -6.3	-4.156	<0.001	**0.0003**
Stimulation voltage	+0.0 (0.0)	-0.02 to +0.03	0.303	0.762	1.0000
Body mass index	-1.0 (1.7)	-4.4 to +2.4	-0.580	0.562	1.0000
Age	+0.1 (0.8)	-1.5 to +1.6	-0.088	0.930	1.0000
DBP (mmHg), Coeff. (SE)					
Week 4 vs BL	-5.8 (2.0)	-9.6 to -1.9	-2.914	0.004	**0.0357**
Week 8 vs BL	-6.1 (2.0)	-10.0 to -2.2	-3.080	0.002	**0.0207**
Week 12 vs BL	-6.3 (2.0)	-10.1 to -2.4	-3.162	0.002	**0.0157**
Stimulation voltage	-0.0 (0.0)	-0.01 to + 0.01	-0.072	0.942	1.0000
Body mass index	-0.4 (0.8)	-1.87 to + 1.17	-0.456	0.648	1.0000
Age	-0.0 (0.4)	-0.71 to + 0.66	-0.062	0.950	1.0000
MAP (mmHg), Coeff. (SE)					
Week 4 vs BL	-7.9 (2.2)	-12.1 to -3.6	-3.620	<0.001	**0.0029**
Week 8 vs BL	-7.6 (2.2)	-11.9 to -3.4	-3.518	<0.001	**0.0044**
Week 12 vs BL	-8.2 (2.2)	-12.4 to -3.9	-3.767	<0.001	**0.0017**
Stimulation voltage	+0.0 (0.0)	-0.01 to +0.01	0.132	0.895	1.0000
Body mass index	-0.6 (1.0)	-2.60 to +1.46	-0.549	0.583	1.0000
Age	0.0 (0.5)	-0.90 to +0.92	0.018	0.986	1.0000
HR (beats/min), Coeff. (SE)					
Week 4 vs BL	-0.6 (1.8)	-4.0 to +2.8	-0.340	0.734	1.0000
Week 8 vs BL	-0.4 (1.8)	-3.9 to +3.0	-0.248	0.804	1.0000
Week 12 vs BL	-0.9 (1.8)	-4.4 to +2.5	-0.536	0.592	1.0000
Stimulation voltage	+0.0 (0.0)	-0.00 to +0.02	1.369	0.171	1.0000
Body mass index	+0.7 (0.8)	-0.94 to +2.26	0.806	0.420	1.0000
Age	+0.5 (0.4)	-0.21 to + 1.24	1.402	0.161	1.0000
RMSSD (ms), Coeff. (SE)					
Week 4 vs BL	+1.7 (9.0)	-15.9 to +193	0.190	0.849	1.0000
Week 8 vs BL	-9.2 (9.0)	-26.2 to + 7.9	-1.057	0.291	1.0000
Week 12 vs BL	-12.8 (9.0)	-30.3 to + 4.7	-1.432	0.152	1.0000
Stimulation voltage	+0.0 (0.0)	-0.01 to +0.02	0.805	0.421	1.0000
Body mass index	+3.2 (1.3)	+0.75 to +5.75	2.548	0.011	0.1082
Age	+0.6 (0.6)	-0.56 to +1.65	0.969	0.333	1.0000
Hypertensive without hypertension medication					
SBP (mmHg), Coeff. (SE)					
Week 4 vs BL	-10.4 (3.5)	-17.2 to -3.6	-3.007	0.003	**0.0237**
Week 8 vs BL	-10.6 (3.5)	-17.4 to -3.8	-3.066	0.002	**0.0195**
Week 12 vs BL	-11.0 (3.5)	-17.8 to -4.2	-3.185	0.001	**0.0130**
Stimulation voltage	-0.0 (0.0)	-0.03 to -0.01	-4.216	0.000	**0.0002**
Body mass index	-2.6 (0.7)	-4.04 to -1.17	-3.566	0.000	**0.0089**
Age	+0.7 (0.2)	+0.30 to +1.17	-3.292	0.001	**0.0002**
DBP (mmHg), Coeff. (SE)					
Week 4 vs BL	-5.7 (2.6)	-10.7 to -0.6	-2.211	0.027	0.2434
Week 8 vs BL	-6.0 (2.6)	-11.0 to -1.0	-2.351	0.019	0.1685
Week 12 vs BL	-6.5 (2.6)	-11.6 to -1.5	-2.547	0.011	0.0978

Stimulation voltage	-0.0 (0.0)	-0.02 to -0.00	-2.913	0.004	**0.0322**
Body mass index	-0.5 (0.5)	-1.55 to + 0.58	-0.897	0.369	1.0000
Age	+0.5 (0.2)	+0.14 to +0.79	2.830	0.005	**0.0420**
MAP (mmHg), Coeff. (SE)					
Week 4 vs BL	-7.2 (2.7)	-12.6 to -1.9	-2.677	0.007	0.0669
Week 8 vs BL	-7.6 (2.7)	-12.9 to -2.3	-2.790	0.005	**0.0474**
Week 12 vs BL	-8.0 (2.7)	-13.3 to -2.7	-2.965	0.003	**0.0273**
Stimulation voltage	-0.0 (0.0)	-0.02 to -0.01	-3.635	0.000	**0.0025**
Body mass index	-1.2 (0.6)	-2.31 to -0.07	-2.086	0.037	0.3328
Age	+0.6 (0.2)	+0.21 to +0.90	3.189	0.001	**0.0129**
HR (beats/min), Coeff. (SE)					
Week 4 vs BL	-0.9 (2.9)	-6.5 to +4.8	-0.299	0.765	1.0000
Week 8 vs BL	+0.4 (2.9)	-5.2 to +6.1	0.152	0.879	1.0000
Week 12 vs BL	-0.2 (2.9)	-5.9 to +5.5	-0.071	0.944	1.0000
Stimulation voltage	-0.0 (0.0)	-0.03 to +0.01	-0.796	0.426	1.0000
Body mass index	-2.2 (1.4)	-5.00 to +0.60	-1.539	0.124	1.0000
Age	+0.2 (0.4)	-0.64 to +1.06	0.477	0.633	1.0000
RMSSD (ms), Coeff. (SE)					
Week 4 vs BL	-6.6 (11.0)	-28.3 to +15.0	-0.599	0.549	1.0000
Week 8 vs BL	-1.7 (11.0)	-23.4 to +19.9	-0.154	0.877	1.0000
Week 12 vs BL	-22.0 (11.0)	-43.7 to -0.4	-1.993	0.046	0.4162
Stimulation voltage	+0.0 (0.0)	-0.00 to +0.06	1.871	0.061	0.5519
Body mass index	+7.2 (2.3)	+2.58 to +11.72	3.067	0.002	**0.0195**
Age	+0.5 (0.7)	-0.87 to +1.92	0.739	0.460	1.0000
Hypertensive with hypertension medication					
SBP (mmHg), Coeff. (SE)					
Week 4 vs BL	-14.0 (5.1)	-24.0 to -4.0	-2.744	0.006	0.0547
Week 8 vs BL	-10.8 (5.1)	-20.8 to -0.8	-2.124	0.034	0.3027
Week 12 vs BL	-13.2 (5.1)	-23.2 to -3.2	-2.586	0.010	0.0875
Stimulation voltage	+0.0 (0.0)	-0.01 to +0.03	1.286	0.198	1.0000
Body mass index	10.7 (5.6)	-0.18 to +21.66	1.927	0.054	0.4856
Age	-0.3 (0.8)	-1.77 to +1.25	-0.340	0.734	1.0000
DBP (mmHg), Coeff. (SE)					
Week 4 vs BL	-5.9 (3.3)	-12.4 to +0.6	-1.775	0.076	0.6832
Week 8 vs BL	-6.2 (3.3)	-12.6 to +0.3	-1.862	0.063	0.5630
Week 12 vs BL	-5.9 (3.3)	-12.4 to +0.6	-1.788	0.074	0.6634
Stimulation voltage	+0.0 (0.0)	-0.01 to +0.01	0.115	0.991	1.0000
Body mass index	-0.0 (3.6)	-6.12 to +7.05	-0.011	0.991	1.0000
Age	-0.6 (0.5)	-1.20 to +0.23	-1.204	0.229	1.0000
MAP (mmHg), Coeff. (SE)					
Week 4 vs BL	-8.6 (3.8)	-15.9 to -1.2	-2.288	0.022	0.1994
Week 8 vs BL	-7.7 (3.8)	-15.1 to -0.4	-2.058	0.040	0.3561
Week 12 vs BL	-8.3 (3.8)	-15.7 to -1.0	-2.224	0.026	0.2354
Stimulation voltage	+0.0 (0.0)	-0.01 to +0.02	0.651	0.515	1.0000
Body mass index	+3.6 (4.1)	-4.48 to +11.58	0.867	0.386	1.0000
Age	-0.5 (0.6)	-1.60 to +0.62	-0.862	0.389	1.0000
HR (beats/min), Coeff. (SE)					
Week 4 vs BL	-0.3 (2.0)	-4.1 to +3.6	-0.137	0.891	1.0000
Week 8 vs BL	-1.5 (2.0)	-5.3 to +2.4	-0.756	0.450	1.0000
Week 12 vs BL	-1.8 (2.0)	-5.7 to´+2.0	-0.926	0.354	1.0000
Stimulation voltage	+0.0 (0.0)	+0.01 to +0.02	3.486	0.000	**0.0044**
Body mass index	+3.6 (2.1)	-0.61 to + 7.78	1.675	0.094	0.8447
Age	+0.8 (0.3)	+0.26 to +1.42	2.852	0.004	**0.0391**
RMSSD (ms), Coeff. (SE)					

Week 4 vs BL	+11.9 (12.7)	-12.9 to +36.9	0.942	0.346	1.0000
Week 8 vs BL	-18.2 (11.8)	-41.2 to +4.9	-1.545	0.122	1.0000
Week 12 vs BL	+2.3 (12.7)	-22.7 to +27.1	0.177	0.860	1.0000
Stimulation voltage	+0.0 (0.0)	-0.04 to +0.04	0.091	0.927	1.0000
Body mass index	-1.2 (13.2)	-27.10 to +24.70	-0.092	0.927	1.0000
Age	+0.7 (1.8)	-2.77 to +4.24	0.409	0.682	1.0000

Supplementary Table 2 Results for pairwise comparisons of blood pressure, heart rate and heart rate variability between baseline (BL, week 1), mid of treatment (week 4), end of treatment (week 8) and end of follow up (week 12). Systolic blood pressure (SBP), diastolic blood pressure (DBP), mean arterial pressure (MAP), heart rate (HR), root mean square of successive differences in RR intervals (RMSSD). SE = standard error

	**Δ [mean (SD)]**	**Cohen's d**	**Power (%)**
SBP (mmHg)			
Week 4 vs BL	6.07 (10.47)	0.58	81.0
Week 8 vs BL	6.13 (11.01)	0.56	77.8
Week 12 vs BL	5.54 (10.97)	0.51	69.6
DBP (mmHg)			
Week 4 vs BL	2.44 (6.66)	0.37	43.4
Week 8 vs BL	3.11 (7.76)	0.40	50.2
Week 12 vs BL	2.21 (7.68)	0.29	29.0
MAP (mmHg)			
Week 4 vs BL	3.65 (7.67)	0.48	64.4
Week 8 vs BL	4.12 (8.55)	0.48	65.5
Week 12 vs BL	3.32	0.39	47.9
HR (beats/min)			
Week 4 vs BL	-0.48 (5.00)	-0.17	13.1
Week 8 vs BL	-0.98 (5.93)	-0.17	12.9
Week 12 vs BL	-2.17 (8.05)	-0.27	26.2
RMSSD (ms)			
Week 4 vs BL	-1.84 (22.57)	-0.08	6.8
Week 8 vs BL	2.90 (24.69)	0.12	8.9
Week 12 vs BL	-1.91 (55.59)	-0.03	5.3
	Non-hypertensive		
SBP (mmHg)			
Week 4 vs BL	1.03 (6.42)	0.16	8.9
Week 8 vs BL	2.25 (7.09)	0.32	20.9
Week 12 vs BL	0.08 (7.02)	0.01	5
DBP (mmHg)			
Week 4 vs BL	-0.37 (4.54)	-0.08	6
Week 8 vs BL	0.60 (5.63)	0.11	6.7
Week 12 vs BL	-1.21 (6.05)	-0.2	11.2
MAP (mmHg)			
Week 4 vs BL	0.10 (4.94)	0.02	5.1
Week 8 vs BL	1.15 (5.89)	0.19	10.8
Week 12 vs BL	-0.78 (6.15)	-0.13	7.4
HR (beats/min)			
Week 4 vs BL	-2.05 (2.78)	-0.74	76
Week 8 vs BL	-2.18 (5.53)	-0.4	29.7
Week 12 vs BL	-4.80 (7.96)	-0.6	58.6
RMSSD (ms)			
Week 4 vs BL	-1.43 (22.24)	-0.06	5.6
Week 8 vs BL	-2.42 (25.35)	-0.1	6.4
Week 12 vs BL	-17.32 (70.91)	-0.24	14.2

Hypertensive			
SBP (mmHg)			
Week 4 vs BL	12.03 (11.43)	1.05	93.7
Week 8 vs BL	10.70 (13.27)	0.81	76.3
Week 12 vs BL	12.00 (11.53)	1.04	93.2
DBP (mmHg),			
Week 4 vs BL	5.76 (7.40)	0.78	73.2
Week 8 vs BL	6.08 (9.08)	0.67	60.3
Week 12 vs BL	6.25 (7.66)	0.82	77.1
MAP (mmHg)			
Week 4 vs BL	7.85 (8.39)	0.94	87.4
Week 8 vs BL	7.62 (10.07)	0.76	70.9
Week 12 vs BL	8.16 (8.60)	0.95	88.2
HR (beats/min)			
Week 4 vs BL	0.59 (6.63)	0.09	6
Week 8 vs BL	0.43 (6.33)	0.07	5.6
Week 12 vs BL	0.94 (7.32)	0.13	7.1
RMSSD (ms)			
Week 4 vs BL	-2.33 (24.15)	-0.1	6.2
Week 8 vs BL	9.18 (23.46)	0.39	25.5
Week 12 vs BL	13.51 (31.14)	0.43	30.1
Hypertensive without hypertension medication			
SBP (mmHg)			
Week 4 vs BL	10.40 (9.36)	1.11	77.7
Week 8 vs BL	10.60 (10.89)	0.97	66.4
Week 12 vs BL	11.02 (11.05)	1	68.5
DBP (mmHg)			
Week 4 vs BL	5.66 (6.97)	0.81	51.2
Week 8 vs BL	6.02 (8.62)	0.7	40.2
Week 12 vs BL	6.52 (7.57)	0.86	56.1
MAP (mmHg)			
Week 4 vs BL	7.24 (7.45)	0.97	66.3
Week 8 vs BL	7.55 (8.84)	0.85	55.2
Week 12 vs BL	8.02 (8.30)	0.97	65.9
HR (beats/min)			
Week 4 vs BL	0.86 (8.72)	0.1	5.7
Week 8 vs BL	-0.44 (8.67)	-0.05	5.2
Week 12 vs BL	0.20 (8.73)	0.02	5
RMSSD (ms)			
Week 4 vs BL	6.61 (19.21)	0.34	13.5
Week 8 vs BL	1.71 (24.31)	0.07	5.3
Week 12 vs BL	22.01 (37.22)	0.59	30.5
Hypertensive with hypertension medication			
SBP (mmHg)			
Week 4 vs BL	13.98 (14.44)	0.97	58.1
Week 8 vs BL	10.82 (17.09)	0.63	29.4
Week 12 vs BL	13.17 (13.29)	0.99	60.1
DBP (mmHg)			
Week 4 vs BL	5.87 (8.73)	0.67	32.4
Week 8 vs BL	6.16 (10.63)	0.58	25.3
Week 12 vs BL	5.91 (8.66)	0.68	33.3
MAP (mmHg)			
Week 4 vs BL	8.57 (10.25)	0.84	46.4
Week 8 vs BL	7.71 (12.48)	0.62	28.2

Week 12 vs BL	8.33 (9.94)	0.84	46.6
HR (beats/min)			
Week 4 vs BL	0.27 (3.83)	0.07	5.3
Week 8 vs BL	1.48 (1.95)	0.76	39.6
Week 12 vs BL	1.81 (6.07)	0.3	10.3
RMSSD (ms)			
Week 4 vs BL	-15.75 (27.08)	-0.58	25.5
Week 8 vs BL	18.16 (21.29)	0.85	47.9
Week 12 vs BL	0.75 (15.41)	0.05	5.1

Supplementary Table 3. Post hoc statistical power and effect size estimates for within-group changes in hemodynamic and autonomic parameters across all subgroups at weeks 4, 8, and 12. Δ denotes the unadjusted mean change from baseline (i.e., raw paired differences, not LMM-derived estimates). Cohen's d effect sizes and corresponding statistical power (1−β, two-tailed, α = 0.05) are reported for systolic blood pressure (SBP), diastolic blood pressure (DBP), mean arterial pressure (MAP), heart rate (HR), and root mean square of successive differences (RMSSD). Results confirm adequate power to detect large BP reductions in hypertensive patients—particularly in the untreated subgroup—whereas HR and HRV outcomes exhibited low power in most groups, reflecting small effect sizes and greater interindividual variability. SD = standard deviation.

	**Spearman's Correlation**	**p-value**
SBP BL vs ΔSBP		
Week 4	-0.59	**0.002**
Week 8	-0.33	0.113
Week 12	-0.57	**0.004**
DBP BL vs ΔDBP		
Week 4	-0.60	**0.002**
Week 8	-0.50	**0.012**
Week 12	-0.65	**0.001**
MAP BL vs ΔMAP		
Week 4	-0.61	**0.001**
Week 8	-0.40	0.053
Week 12	-0.64	0.001
HR BL vs ΔSHR		
Week 4	-0.16	0.458
Week 8	-0.63	**0.001**
Week 12	-0.40	0.053
RMSSD BL vs ΔRMSSD		
Week 4	-0.56	**0.006**
Week 8	-0.46	**0.032**
Week 12	-0.71	**0.001**

Supplementary Table 4 Spearman's correlation coefficients and p-values for the relationships between baseline systolic blood pressure (SBP), diastolic blood pressure (DBP), mean arterial pressure (MAP), heart rate (HR), and root mean square of successive differences in RR intervals (RMSSD) with their respective relative percentage changes from baseline (BL, week 1) to mid treatment (week 4), end of treatment (week 8) and end of follow up (week 12).

	**Spearman's correlation**	**p-value**
Voltage vs ΔSBP		
Week 4	-0.03	0.900
Week 8	0.44	0.066
Week 12	0.11	0.663
Voltage vs ΔDBP		

Week 4	0.11	0.669
Week 8	0.22	0.385
Week 12	0.09	0.717
Voltage vs ΔMAP		
Week 4	-0.05	0.848
Week 8	0.27	0.287
Week 12	0.11	0.651
Voltage vs ΔHR		
Week 4	-0.54	0.021
Week 8	-0.39	0.106
Week 12	-0.31	0.203
Voltage vs ΔRMSSD		
Week 4	-0.23	0.363
Week 8	0.26	0.299
Week 12	-0.01	0.971

Supplementary Table 5 Spearman's correlation coefficients and p-values for the relationships between stimulation voltage and the respective changes in systolic blood pressure (SBP), diastolic blood pressure (DBP), mean arterial pressure (MAP), heart rate (HR), and root mean square of successive differences in RR intervals (RMSSD) from baseline (BL, week 1) to mid treatment (week 4), end of treatment (week 8) and end of follow up (week 12).

	**Baseline**	**Week 4**	**Week 8**	**Week 12**
Non-hypertensive				
SBP (mmHg), mean (SD)	123.9 (10.1)	122.8 (10.1)	121.6 (11.4)	123.8 (10.2)
95% CI	118.4 to 129.3	118.4 to 129.3	115.4 to 127.8	118.2 to 129.3
DBP (mmHg), mean (SD)	80.2 (6.5)	80.6 (5.3)	79.6 (6.0)	81.4 (6.0)
95% CI	76.7 to 83.8	77.7 to 83.5	76.4 to 82.9	77.9 to 85.0
MAP (mmHg)	94.8 (7.4)	94.7 (5.8)	93.6 (7.3)	95.6 (7.1)
95% CI	90.8 to 98.8	91.5 to 97.9	89.6 to 97.6	91.7 to 99.4
Heart rate (beats/min), mean (SD)	75.3 (7.6)	77.3 (8.7)	77.4 (7.8)	80.1 (9.6)
95% CI	71.1 to 79.4	72.6 to 82.0	73.2 to 81.7	74.8 to 85.3
RMSSD (ms), mean (SD)	62.0 (19.2)	61.9 (18.9)	64.5 (22.6)	73.5 (76.5)
95% CI	51.6 to 72.4	51.3 to 72.6	52.2 to 76.7	26.1 to 120.9
Hypertensive				
SBP (mmHg), mean (SD)	151.0 (10.3)	139.0 (11.9)	140.3 (15.0)	139.0 (12.8)
95% CI	144.9 to 157.1	132.0 to 146.0	131.5 to 149.2	131.5 to 146.6
DBP (mmHg), mean (SD)	98.3 (3.7)	92.5 (6.7)	92.2 (8.2)	92.1 (6.3)
95% CI	96.1 to 100.5	88.6 to 86.5	87.4 to 97.1	88.3 to 95.8
MAP (mmHg)	115.9 (5.1)	108.0 (8.1)	108.3 (10.1)	107.7 (7.7)
95% CI	112.9 to 118.9	103.3 to 112.8	102.3 to 114.2	103.1 to 112.3
Heart rate (beats/min), mean (SD)	79.4 (6.9)	78.8 (7.7)	79.0 (5.2)	78.5 (8.6)
95% CI	75.3 to 83.5	74.3 to 83.4	75.9 to 82.1	73.4 to 83.6
RMSSD (ms), mean (SD)	63.9 (21.5)	64.8 (20.9)	54.7 (23.3)	51.3 (19.8)
95% CI	51.2 to 76.6	51.8 to 77.7	40.9 to 68.5	39.0 to 63.5

Supplementary Table 6 Evolvement of blood pressure (BP), heart rate and heart rate variability from study start to end of follow up. Data are presented as mean (standard deviation) for systolic blood pressure (SBP), diastolic blood pressure (DBP), mean arterial pressure (MAP), heart rate and root mean square of successive differences in normal RR-intervals (RMSSD) at baseline (week 1), mid of treatment (week 4), and end of treatment (week 8) and end of follow up (week 12). CI, confidence interval; SD standard deviation. Note: For statistical comparisons, including p-values and effect sizes (Cohen's d) for within-group changes over time, see **Supplementary Table 3.**

S1. Subgroup analysis of treated and untreated hypertensive patients

Untreated hypertensive patients exhibited significant reductions in systolic and mean arterial pressure over time. From baseline to week 12, SBP decreased by 11.0 (11.05) mmHg ($p = 0.001$), MAP by 8.02 (8.30) mmHg ($p = 0.003$), and DBP by 6.52 (7.57) mmHg ($p = 0.011$). These changes corresponded to large within-group effect sizes (Cohen's

d = 1.00, 0.97, and 0.86, respectively), with post hoc power estimates exceeding 65% across all parameters. Heart rate remained unchanged, with a minimal change from 77.5 (8.1) bpm at baseline to 77.3 (8.2) bpm at week 12 (Δ = 0.20 bpm, p = 0.944, d = 0.02). Heart rate variability, measured via RMSSD, declined modestly from 70.3 (26.1) ms to 48.3 (25.5) ms (Δ = −22.0 ms, p = 0.046, d = 0.59), though this change did not remain statistically significant after correction. These results are visualized in Figure 4 and detailed in Supplementary Tables 2 and 5. By contrast, patients receiving pharmacological treatment also showed reductions in BP (e.g., SBP: −13.2 mmHg at week 12), but these did not reach statistical significance (all p > 0.05), likely due to higher baseline variability or medication-related confounding. HR and RMSSD remained stable across time points. Taken together, these findings suggest that aVNS led to clinically meaningful and statistically significant BP reductions in untreated patients, with effects persisting into follow-up. In contrast, BP reductions in treated hypertensives were more variable and did not achieve statistical significance. HR and HRV remained largely unchanged across all groups, indicating that the BP-lowering effects of aVNS occurred without broader alterations in autonomic tone. Supplementary Figure 2 illustrates individual trajectories in BP and HR, highlighting interindividual variability across subgroups.

In the subgroup of untreated hypertensive patients, stimulation voltage was significantly associated with reductions in SBP (β = –0.02 mmHg/mV, p < 0.001) and MAP (β = –0.01 mmHg/mV, p < 0.001), indicating a dose–response effect of stimulation intensity. Similarly, age and BMI emerged as significant covariates in some subgroup models. For instance, BMI was positively associated with SBP in the treated hypertensive subgroup (p = 0.034), while age showed a modest positive association with DBP in non-hypertensive patients (p = 0.045). These findings suggest that demographic factors may modulate the response to aVNS and merit further investigation in larger, powered studies. Full fixed-effect estimates for all subgroup-level models are provided in Supplementary Table 4.

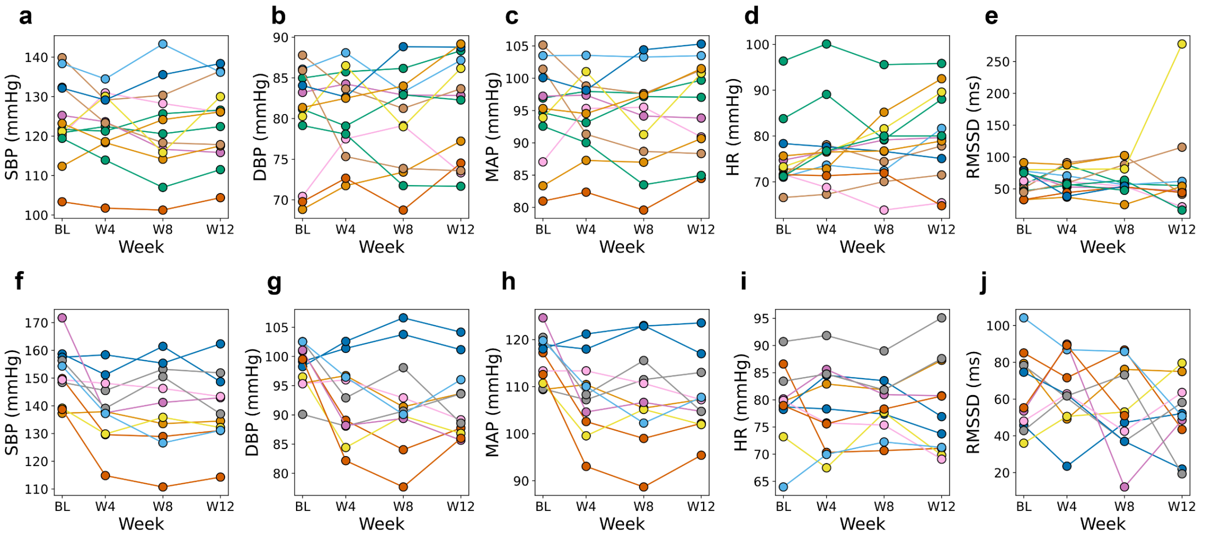

Supplementary Figure 1 Individual trajectories of systolic blood pressure (SBP), diastolic blood pressure (DBP), mean arterial pressure (MAP), heart rate (HR), and root mean square of successive differences (RMSSD) across baseline (BL, week 1), mid of treatment (week 4), end of treatment (week 8), and end of follow up (week 12) in non-hypertensive (**a-e**) and blood pressure (BP) reductions were observed in hypertensive chronic pain patients undergoing auricular vagus nerve stimulation (aVNS). (**f-j**) patients. Each colored line represents an individual subject, illustrating variability in responses to aVNS over time.

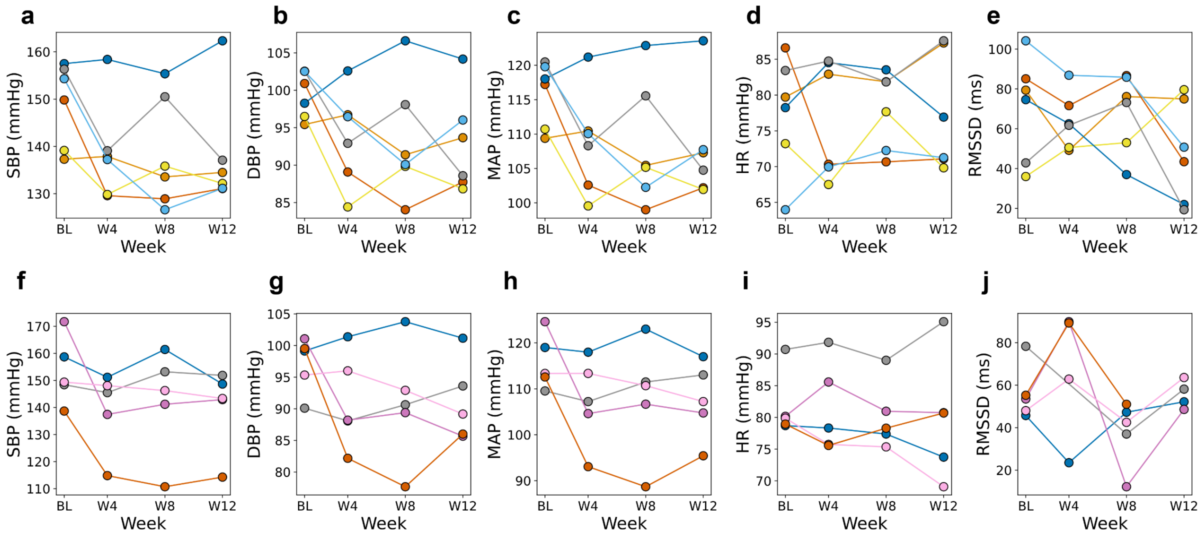

Supplementary Figure 2 Individual trajectories of systolic blood pressure (SBP), diastolic blood pressure (DBP), mean arterial pressure (MAP), heart rate (HR), and root mean square of successive differences in RR intervals (RMSSD) across baseline (BL, week 1), mid of treatment (week 4), end of treatment (week 8), and end of follow up (week 12) in hypertensive patients without hypertension medication (**a-e**) and hypertensive patients with hypertension medication (**f-j**) Each colored line represents an individual subject, illustrating variability in responses to aVNS over time.
