# Supplementary material for: Sustained blood pressure reduction associated with percutaneous auricular vagus nerve stimulation in hypertensive chronic pain patients: a retrospective dual-center analysis
Source: Front Cardiovasc Med. 2026 Mar 30;13:1736774. doi: 10.3389/fcvm.2026.1736774 (PMC13070907; doi:10.3389/fcvm.2026.1736774)
Supplement: Supplementary file 2 [file Table1.docx]

**Supplementary material**

| **Inclusion criteria:** | - Male or female aged ≥ 18 and ≤ 65 years at screening - Patient has an understanding of the study and its procedures, agrees to its provisions, and gives written informed consent prior to any study-related procedures - Chronic back pain with or without leg pain (CBLP) persisting for at least 3 months - A minimum of 70 (out of 180) points on the Computer User Self-Efficacy Scale at the screening visit - Patient is constant with respect to pain treatment during the screening phase - A daily average visual analog scale score (VAS) ≥ 4 on at least half of the days in the screening phase - Compliance with the daily status reporting requirements as demonstrated by having valid required data entries for all days. It is acceptable if data of one day is missing in the screening phase |
| --- | --- |
| **Exclusion criteria:** | - Patients with age under 18 - Hemophilia - Infection, eczema, or psoriasis at application site - Numbed and desensitized skin at the application site - Florid malignant diseases - Mental and physical impairments that represent a source of risk for handling the device - The presence of a cardiac pacemaker, defibrillator, cochlear implant, or other active implantable device - Vagal hypersensitivity - Indication for back surgery - High-grade spinal stenosis - Patients with other active implants - Patients with autonomic disorders - Patients with diabetes type I or II - Patients taking Beta-Blockers - Patients taking drugs carrying the potential risk of arrhythmia (tricyclic medications, Alzheimer drugs, etc.) - Clinically significant hip or knee arthritis - Allergy against rescue medication used during the study - History of Vagus Nerve Stimulation - Pregnant or nursing female patients - Patients with arrhythmia, bradycardia, other rhythm disorders or any other clinically significant cardiac anomalies detected during ECG at screening |

**Supplementary Table 1** Overview of inclusion and exclusion criteria.

|  | **Effect size** | **95% CI** | **z** | **p-value^a^** | **p-value corrected^b^** |
| --- | --- | --- | --- | --- | --- |
| All | | | | | |
| SBP (mmHg), Coeff. (SE) |  |  |  |  |  |
| Week 4 vs BL | -6.1 (1.8) | -9.6 to -2.6 | -3.418 | 0.001 | **0.0069** |
| Week 8 vs BL | -6.1 (1.8) | -9.6 to -2.7 | -3.450 | 0.001 | **0.0062** |
| Week 12 vs BL | -5.5 (1.8) | -9.0 to -2.1 | -3.121 | 0.002 | **0.0199** |
| Stimulation voltage | +0.0 (0.0) | -0.01 to +0.02 | 0.808 | 0.419 | 1.0000 |
| Body mass index | +0.4 (0.6) | -0.87 to +1.65 | 0.602 | 0.547 | 1.0000 |
| Age | +0.2 (0.3) | -0.36 to +0.71 | 0.650 | 0.516 | 1.0000 |
| DBP (mmHg), Coeff. (SE) |  |  |  |  |  |
| Week 4 vs BL | -2.4 (1.2) | -4.8 to -0.1 | 0.284 | 0.046 | 0.5045 |
| Week 8 vs BL | -3.1 (1.2) | -5.5 to -0.7 | -0.458 | 0.011 | 0.1191 |
| Week 12 vs BL | -2.2 (1.2) | -4.6 to 0.2 | 0.929 | 0.071 | 0.7782 |
| Stimulation voltage | -0.0 (0.0) | -0.01 to +0.01 | -0.008 | 0.994 | 1.0000 |
| Body mass index | +0.2 (0.3) | -0.43 to +0.89 | 0.686 | 0.493 | 1.0000 |
| Age | +0.1 (0.1) | -0.20 to +0.36 | 0.615 | 0.538 | 1.0000 |
| MAP (mmHg), Coeff. (SE) |  |  |  |  |  |
| Week 4 vs BL | -3.7 (1.3) | -6.3 to -1.0 | -2.721 | 0.006 | 0.0715 |
| Week 8 vs BL | -4.1 (1.3) | -6.7 to -1.5 | -3.071 | 0.002 | **0.0235** |
| Week 12 vs BL | -3.3 (1.3) | -6.0 to -0.7 | -2.475 | 0.013 | 0.1465 |
| Stimulation voltage | +0.0 (0.0) | -0.01 to +0.01 | 0.421 | 0.674 | 1.0000 |
| Body mass index | +0.3 (0.4) | -0.42 to +1.08 | 0.694 | 0.488 | 1.0000 |
| Age | +0.1 (0.2) | -0.22 to +0.45 | 0.680 | 0.496 | 1.0000 |
| HR (beats/min), Coeff. (SE) |  |  |  |  |  |
| Week 4 vs BL | +0.8 (1.2) | -1.5 to +3.2 | 0.691 | 0.490 | 1.0000 |
| Week 8 vs BL | +1.0 (1.2) | -1.4 to + 3.4 | 0.810 | 0.418 | 1.0000 |
| Week 12 vs BL | +2.2 (1.2) | -0.2 to + 4.6 | 1.787 | 0.074 | 0.8131 |
| Stimulation voltage | +0.0 (0.0) | -0.01 to +0.01 | 0.252 | 0.801 | 1.0000 |
| Body mass index | -0.2 (0.4) | -1.09 to +0.61 | -0.551 | 0.582 | 1.0000 |
| Age | -0.1 (0.2) | -0.43 to +0.29 | -0.397 | 0.691 | 1.0000 |
| RMSSD (ms), Coeff. (SE) |  |  |  |  |  |
| Week 4 vs BL | +2.5 (8.4) | -14.0 to +19.0 | 0.297 | 0.767 | 1.0000 |
| Week 8 vs BL | -2.9 (8.2) | -18.9 to +13.1 | -0.354 | 0.723 | 1.0000 |
| Week 12 vs BL | +0.1 (8.7) | -16.9 to +17.1 | 0.008 | 0.994 | 1.0000 |
| Stimulation voltage | -0.0 (0.0) | -0.04 to +0.02 | -0.486 | 0.627 | 1.0000 |
| Body mass index | +1.4 (1.4) | -1.34 to +4.10 | 0.997 | 0.319 | 1.0000 |
| Age | -0.5 (0.6) | -1.67 to +0.65 | 0.393 | 0.393 | 1.0000 |
| Non-hypertensive | | | | | |
| SBP (mmHg), Coeff. (SE) |  |  |  |  |  |
| Week 4 vs BL | -1.0 (1.7) | -4.4 to +2.4 | -0.590 | 0.555 | 1.0000 |
| Week 8 vs BL | -1.3 (1.7) | -5.7 to +1.2 | -1.294 | 0.196 | 1.0000 |
| Week 12 vs BL | -0.1 (1.7) | -3.5 to +3.3 | -0.044 | 0.965 | 1.0000 |
| Stimulation voltage | +0.0 (0.0) | -0.03 to +0.05 | 0.375 | 0.708 | 1.0000 |
| Body mass index | +1.1 (0.9) | -0.6 to +2.8 | 0.129 | 0.195 | 1.0000 |
| Age | -0.0 (0.4) | -0.8 to +0.8 | -0.007 | 0.995 | 1.0000 |
| DBP (mmHg), Coeff. (SE) |  |  |  |  |  |
| Week 4 vs BL | +0.4 (1.3) | -2.2 to +2.9 | 0.284 | 0.777 | 1.0000 |
| Week 8 vs BL | -0.6 (1.3) | -3.2 to +2.0 | -0.458 | 0.647 | 1.0000 |
| Week 12 vs BL | +1.2 (1.3) | -1.3 to +3.8 | -0.929 | 0.353 | 1.0000 |
| MAP (mmHg), Coeff. (SE) |  |  |  |  |  |
| Week 4 vs BL | -0.1 (1.4) | -2.8 to +2.6 | -0.071 | 0.944 | 1.0000 |
| Week 8 vs BL | -1.2 (1.4) | -3.8 to +1.5 | -0.849 | 0.396 | 1.0000 |
| Week 12 vs BL | +0.8 (1.4) | -1.9 to +3.4 | 0.578 | 0.563 | 1.0000 |
| Stimulation voltage | +0.0 (0.0) | -0.02 to +0.03 | 0.251 | 0.802 | 1.0000 |
| Body mass index | +0.8 (0.6) | -0.24 to +1.94 | 1.524 | 0.128 | 1.0000 |
| Age | +0.0 (0.3) | -0.49 to +0.50 | 0.030 | 0.976 | 1.0000 |
| HR (beats/min), Coeff. (SE) |  |  |  |  |  |
| Week 4 vs BL | +2.1 (1.6) | -1.2 to +5.3 | 1.258 | 0.208 | 1.0000 |
| Week 8 vs BL | +2.2 (1.6) | -1.0 to +5.4 | 1.338 | 0.181 | 1.0000 |
| Week 12 vs BL | +4.8 (1.6) | +1.6 to +8.0 | 2.943 | 0.003 | **0.0292** |
| Stimulation voltage | -0.0 (0.0) | -0.04 to +0.03 | -0.057 | 0.954 | 1.0000 |
| Body mass index | -0.3 (0.8) | -1.25 to +1.90 | 0.403 | 0.687 | 1.0000 |
| Age | -0.3 (0.4) | -1.02 to +0.40 | -0.849 | 0.397 | 1.0000 |
| RMSSD (ms), Coeff. (SE) |  |  |  |  |  |
| Week 4 vs BL | +4.5 (13.6) | -22.2 to +31.3 | 0.331 | 0.740 | 1.0000 |
| Week 8 vs BL | +2.4 (13.3) | -23.5 to +28.4 | 0.183 | 0.855 | 1.0000 |
| Week 12 vs BL | +11.9 (14.5) | -16.4 to +40.3 | 0.825 | 0.410 | 1.0000 |
| Stimulation voltage | -0.0 (0.1) | -0.14 to +0.12 | -0.142 | 0.887 | 1.0000 |
| Body mass index | +1.7 (2.9) | -4.03 to +7.50 | 0.590 | 0.555 | 1.0000 |
| Age | -0.7 (1.3) | -3.27 to +2.00 | -0.486 | 0.627 | 1.0000 |
| Hypertensive | | | | | |
| SBP (mmHg), Coeff. (SE) |  |  |  |  |  |
| Week 4 vs BL | -12.0 (2.9) | -17.7 to -6.4 | -4.167 | <0.001 | **0.0003** |
| Week 8 vs BL | -10.7 (2.9) | -16.4 to +5.1 | -3.708 | <0.001 | **0.0021** |
| Week 12 vs BL | -12.0 (2.9) | -17.7 to -6.3 | -4.156 | <0.001 | **0.0003** |
| Stimulation voltage | +0.0 (0.0) | -0.02 to +0.03 | 0.303 | 0.762 | 1.0000 |
| Body mass index | -1.0 (1.7) | -4.4 to +2.4 | -0.580 | 0.562 | 1.0000 |
| Age | +0.1 (0.8) | -1.5 to +1.6 | -0.088 | 0.930 | 1.0000 |
| DBP (mmHg), Coeff. (SE) |  |  |  |  |  |
| Week 4 vs BL | -5.8 (2.0) | -9.6 to -1.9 | -2.914 | 0.004 | **0.0357** |
| Week 8 vs BL | -6.1 (2.0) | -10.0 to -2.2 | -3.080 | 0.002 | **0.0207** |
| Week 12 vs BL | -6.3 (2.0) | -10.1 to -2.4 | -3.162 | 0.002 | **0.0157** |
| Stimulation voltage | -0.0 (0.0) | -0.01 to + 0.01 | -0.072 | 0.942 | 1.0000 |
| Body mass index | -0.4 (0.8) | -1.87 to + 1.17 | -0.456 | 0.648 | 1.0000 |
| Age | -0.0 (0.4) | -0.71 to + 0.66 | -0.062 | 0.950 | 1.0000 |
| MAP (mmHg), Coeff. (SE) |  |  |  |  |  |
| Week 4 vs BL | -7.9 (2.2) | -12.1 to -3.6 | -3.620 | <0.001 | **0.0029** |
| Week 8 vs BL | -7.6 (2.2) | -11.9 to -3.4 | -3.518 | <0.001 | **0.0044** |
| Week 12 vs BL | -8.2 (2.2) | -12.4 to -3.9 | -3.767 | <0.001 | **0.0017** |
| Stimulation voltage | +0.0 (0.0) | -0.01 to +0.01 | 0.132 | 0.895 | 1.0000 |
| Body mass index | -0.6 (1.0) | -2.60 to +1.46 | -0.549 | 0.583 | 1.0000 |
| Age | 0.0 (0.5) | -0.90 to +0.92 | 0.018 | 0.986 | 1.0000 |
| HR (beats/min), Coeff. (SE) |  |  |  |  |  |
| Week 4 vs BL | -0.6 (1.8) | -4.0 to +2.8 | -0.340 | 0.734 | 1.0000 |
| Week 8 vs BL | -0.4 (1.8) | -3.9 to +3.0 | -0.248 | 0.804 | 1.0000 |
| Week 12 vs BL | -0.9 (1.8) | -4.4 to +2.5 | -0.536 | 0.592 | 1.0000 |
| Stimulation voltage | +0.0 (0.0) | -0.00 to +0.02 | 1.369 | 0.171 | 1.0000 |
| Body mass index | +0.7 (0.8) | -0.94 to +2.26 | 0.806 | 0.420 | 1.0000 |
| Age | +0.5 (0.4) | -0.21 to + 1.24 | 1.402 | 0.161 | 1.0000 |
| RMSSD (ms), Coeff. (SE) |  |  |  |  |  |
| Week 4 vs BL | +1.7 (9.0) | -15.9 to +193 | 0.190 | 0.849 | 1.0000 |
| Week 8 vs BL | -9.2 (9.0) | -26.2 to + 7.9 | -1.057 | 0.291 | 1.0000 |
| Week 12 vs BL | -12.8 (9.0) | -30.3 to + 4.7 | -1.432 | 0.152 | 1.0000 |
| Stimulation voltage | +0.0 (0.0) | -0.01 to +0.02 | 0.805 | 0.421 | 1.0000 |
| Body mass index | +3.2 (1.3) | +0.75 to +5.75 | 2.548 | 0.011 | 0.1082 |
| Age | +0.6 (0.6) | -0.56 to +1.65 | 0.969 | 0.333 | 1.0000 |
| Hypertensive without hypertension medication | | | | | |
| SBP (mmHg), Coeff. (SE) |  |  |  |  |  |
| Week 4 vs BL | -10.4 (3.5) | -17.2 to -3.6 | -3.007 | 0.003 | **0.0237** |
| Week 8 vs BL | -10.6 (3.5) | -17.4 to -3.8 | -3.066 | 0.002 | **0.0195** |
| Week 12 vs BL | -11.0 (3.5) | -17.8 to -4.2 | -3.185 | 0.001 | **0.0130** |
| Stimulation voltage | -0.0 (0.0) | -0.03 to -0.01 | -4.216 | 0.000 | **0.0002** |
| Body mass index | -2.6 (0.7) | -4.04 to -1.17 | -3.566 | 0.000 | **0.0089** |
| Age | +0.7 (0.2) | +0.30 to +1.17 | -3.292 | 0.001 | **0.0002** |
| DBP (mmHg), Coeff. (SE) |  |  |  |  |  |
| Week 4 vs BL | -5.7 (2.6) | -10.7 to -0.6 | -2.211 | 0.027 | 0.2434 |
| Week 8 vs BL | -6.0 (2.6) | -11.0 to -1.0 | -2.351 | 0.019 | 0.1685 |
| Week 12 vs BL | -6.5 (2.6) | -11.6 to -1.5 | -2.547 | 0.011 | 0.0978 |
| Stimulation voltage | -0.0 (0.0) | -0.02 to -0.00 | -2.913 | 0.004 | **0.0322** |
| Body mass index | -0.5 (0.5) | -1.55 to + 0.58 | -0.897 | 0.369 | 1.0000 |
| Age | +0.5 (0.2) | +0.14 to +0.79 | 2.830 | 0.005 | **0.0420** |
| MAP (mmHg), Coeff. (SE) |  |  |  |  |  |
| Week 4 vs BL | -7.2 (2.7) | -12.6 to -1.9 | -2.677 | 0.007 | 0.0669 |
| Week 8 vs BL | -7.6 (2.7) | -12.9 to -2.3 | -2.790 | 0.005 | **0.0474** |
| Week 12 vs BL | -8.0 (2.7) | -13.3 to -2.7 | -2.965 | 0.003 | **0.0273** |
| Stimulation voltage | -0.0 (0.0) | -0.02 to -0.01 | -3.635 | 0.000 | **0.0025** |
| Body mass index | -1.2 (0.6) | -2.31 to -0.07 | -2.086 | 0.037 | 0.3328 |
| Age | +0.6 (0.2) | +0.21 to +0.90 | 3.189 | 0.001 | **0.0129** |
| HR (beats/min), Coeff. (SE) |  |  |  |  |  |
| Week 4 vs BL | -0.9 (2.9) | -6.5 to +4.8 | -0.299 | 0.765 | 1.0000 |
| Week 8 vs BL | +0.4 (2.9) | -5.2 to +6.1 | 0.152 | 0.879 | 1.0000 |
| Week 12 vs BL | -0.2 (2.9) | -5.9 to +5.5 | -0.071 | 0.944 | 1.0000 |
| Stimulation voltage | -0.0 (0.0) | -0.03 to +0.01 | -0.796 | 0.426 | 1.0000 |
| Body mass index | -2.2 (1.4) | -5.00 to +0.60 | -1.539 | 0.124 | 1.0000 |
| Age | +0.2 (0.4) | -0.64 to +1.06 | 0.477 | 0.633 | 1.0000 |
| RMSSD (ms), Coeff. (SE) |  |  |  |  |  |
| Week 4 vs BL | -6.6 (11.0) | -28.3 to +15.0 | -0.599 | 0.549 | 1.0000 |
| Week 8 vs BL | -1.7 (11.0) | -23.4 to +19.9 | -0.154 | 0.877 | 1.0000 |
| Week 12 vs BL | -22.0 (11.0) | -43.7 to -0.4 | -1.993 | 0.046 | 0.4162 |
| Stimulation voltage | +0.0 (0.0) | -0.00 to +0.06 | 1.871 | 0.061 | 0.5519 |
| Body mass index | +7.2 (2.3) | +2.58 to +11.72 | 3.067 | 0.002 | **0.0195** |
| Age | +0.5 (0.7) | -0.87 to +1.92 | 0.739 | 0.460 | 1.0000 |
| Hypertensive with hypertension medication | | | | | |
| SBP (mmHg), Coeff. (SE) |  |  |  |  |  |
| Week 4 vs BL | -14.0 (5.1) | -24.0 to -4.0 | -2.744 | 0.006 | 0.0547 |
| Week 8 vs BL | -10.8 (5.1) | -20.8 to -0.8 | -2.124 | 0.034 | 0.3027 |
| Week 12 vs BL | -13.2 (5.1) | -23.2 to -3.2 | -2.586 | 0.010 | 0.0875 |
| Stimulation voltage | +0.0 (0.0) | -0.01 to +0.03 | 1.286 | 0.198 | 1.0000 |
| Body mass index | 10.7 (5.6) | -0.18 to +21.66 | 1.927 | 0.054 | 0.4856 |
| Age | -0.3 (0.8) | -1.77 to +1.25 | -0.340 | 0.734 | 1.0000 |
| DBP (mmHg), Coeff. (SE) |  |  |  |  |  |
| Week 4 vs BL | -5.9 (3.3) | -12.4 to +0.6 | -1.775 | 0.076 | 0.6832 |
| Week 8 vs BL | -6.2 (3.3) | -12.6 to +0.3 | -1.862 | 0.063 | 0.5630 |
| Week 12 vs BL | -5.9 (3.3) | -12.4 to +0.6 | -1.788 | 0.074 | 0.6634 |
| Stimulation voltage | +0.0 (0.0) | -0.01 to +0.01 | 0.115 | 0.991 | 1.0000 |
| Body mass index | -0.0 (3.6) | -6.12 to +7.05 | -0.011 | 0.991 | 1.0000 |
| Age | -0.6 (0.5) | -1.20 to +0.23 | -1.204 | 0.229 | 1.0000 |
| MAP (mmHg), Coeff. (SE) |  |  |  |  |  |
| Week 4 vs BL | -8.6 (3.8) | -15.9 to -1.2 | -2.288 | 0.022 | 0.1994 |
| Week 8 vs BL | -7.7 (3.8) | -15.1 to -0.4 | -2.058 | 0.040 | 0.3561 |
| Week 12 vs BL | -8.3 (3.8) | -15.7 to -1.0 | -2.224 | 0.026 | 0.2354 |
| Stimulation voltage | +0.0 (0.0) | -0.01 to +0.02 | 0.651 | 0.515 | 1.0000 |
| Body mass index | +3.6 (4.1) | -4.48 to +11.58 | 0.867 | 0.386 | 1.0000 |
| Age | -0.5 (0.6) | -1.60 to +0.62 | -0.862 | 0.389 | 1.0000 |
| HR (beats/min), Coeff. (SE) |  |  |  |  |  |
| Week 4 vs BL | -0.3 (2.0) | -4.1 to +3.6 | -0.137 | 0.891 | 1.0000 |
| Week 8 vs BL | -1.5 (2.0) | -5.3 to +2.4 | -0.756 | 0.450 | 1.0000 |
| Week 12 vs BL | -1.8 (2.0) | -5.7 to´+2.0 | -0.926 | 0.354 | 1.0000 |
| Stimulation voltage | +0.0 (0.0) | +0.01 to +0.02 | 3.486 | 0.000 | **0.0044** |
| Body mass index | +3.6 (2.1) | -0.61 to + 7.78 | 1.675 | 0.094 | 0.8447 |
| Age | +0.8 (0.3) | +0.26 to +1.42 | 2.852 | 0.004 | **0.0391** |
| RMSSD (ms), Coeff. (SE) |  |  |  |  |  |
| Week 4 vs BL | +11.9 (12.7) | -12.9 to +36.9 | 0.942 | 0.346 | 1.0000 |
| Week 8 vs BL | -18.2 (11.8) | -41.2 to +4.9 | -1.545 | 0.122 | 1.0000 |
| Week 12 vs BL | +2.3 (12.7) | -22.7 to +27.1 | 0.177 | 0.860 | 1.0000 |
| Stimulation voltage | +0.0 (0.0) | -0.04 to +0.04 | 0.091 | 0.927 | 1.0000 |
| Body mass index | -1.2 (13.2) | -27.10 to +24.70 | -0.092 | 0.927 | 1.0000 |
| Age | +0.7 (1.8) | -2.77 to +4.24 | 0.409 | 0.682 | 1.0000 |

**Supplementary Table 2** Results for pairwise comparisons of blood pressure, heart rate and heart rate variability between baseline (BL, week 1), mid of treatment (week 4), end of treatment (week 8) and end of follow up (week 12). Systolic blood pressure (SBP), diastolic blood pressure (DBP), mean arterial pressure (MAP), heart rate (HR), root mean square of successive differences in RR intervals (RMSSD). SE = standard error

|  | **Δ [mean (SD)]** | **Cohen's d** | **Power (%)** |
| --- | --- | --- | --- |
| SBP (mmHg) |  |  |  |
| Week 4 vs BL | 6.07 (10.47) | 0.58 | 81.0 |
| Week 8 vs BL | 6.13 (11.01) | 0.56 | 77.8 |
| Week 12 vs BL | 5.54 (10.97) | 0.51 | 69.6 |
| DBP (mmHg) |  |  |  |
| Week 4 vs BL | 2.44 (6.66) | 0.37 | 43.4 |
| Week 8 vs BL | 3.11 (7.76) | 0.40 | 50.2 |
| Week 12 vs BL | 2.21 (7.68) | 0.29 | 29.0 |
| MAP (mmHg) |  |  |  |
| Week 4 vs BL | 3.65 (7.67) | 0.48 | 64.4 |
| Week 8 vs BL | 4.12 (8.55) | 0.48 | 65.5 |
| Week 12 vs BL | 3.32 | 0.39 | 47.9 |
| HR (beats/min) |  |  |  |
| Week 4 vs BL | -0.48 (5.00) | -0.17 | 13.1 |
| Week 8 vs BL | -0.98 (5.93) | -0.17 | 12.9 |
| Week 12 vs BL | -2.17 (8.05) | -0.27 | 26.2 |
| RMSSD (ms) |  |  |  |
| Week 4 vs BL | -1.84 (22.57) | -0.08 | 6.8 |
| Week 8 vs BL | 2.90 (24.69) | 0.12 | 8.9 |
| Week 12 vs BL | -1.91 (55.59) | -0.03 | 5.3 |
| Non-hypertensive | | | |
| SBP (mmHg) |  |  |  |
| Week 4 vs BL | 1.03 (6.42) | 0.16 | 8.9 |
| Week 8 vs BL | 2.25 (7.09) | 0.32 | 20.9 |
| Week 12 vs BL | 0.08 (7.02) | 0.01 | 5 |
| DBP (mmHg) |  |  |  |
| Week 4 vs BL | -0.37 (4.54) | -0.08 | 6 |
| Week 8 vs BL | 0.60 (5.63) | 0.11 | 6.7 |
| Week 12 vs BL | -1.21 (6.05) | -0.2 | 11.2 |
| MAP (mmHg) |  |  |  |
| Week 4 vs BL | 0.10 (4.94) | 0.02 | 5.1 |
| Week 8 vs BL | 1.15 (5.89) | 0.19 | 10.8 |
| Week 12 vs BL | -0.78 (6.15) | -0.13 | 7.4 |
| HR (beats/min) |  |  |  |
| Week 4 vs BL | -2.05 (2.78) | -0.74 | 76 |
| Week 8 vs BL | -2.18 (5.53) | -0.4 | 29.7 |
| Week 12 vs BL | -4.80 (7.96) | -0.6 | 58.6 |
| RMSSD (ms) |  |  |  |
| Week 4 vs BL | -1.43 (22.24) | -0.06 | 5.6 |
| Week 8 vs BL | -2.42 (25.35) | -0.1 | 6.4 |
| Week 12 vs BL | -17.32 (70.91) | -0.24 | 14.2 |
| Hypertensive | | | |
| SBP (mmHg) |  |  |  |
| Week 4 vs BL | 12.03 (11.43) | 1.05 | 93.7 |
| Week 8 vs BL | 10.70 (13.27) | 0.81 | 76.3 |
| Week 12 vs BL | 12.00 (11.53) | 1.04 | 93.2 |
| DBP (mmHg), |  |  |  |
| Week 4 vs BL | 5.76 (7.40) | 0.78 | 73.2 |
| Week 8 vs BL | 6.08 (9.08) | 0.67 | 60.3 |
| Week 12 vs BL | 6.25 (7.66) | 0.82 | 77.1 |
| MAP (mmHg) |  |  |  |
| Week 4 vs BL | 7.85 (8.39) | 0.94 | 87.4 |
| Week 8 vs BL | 7.62 (10.07) | 0.76 | 70.9 |
| Week 12 vs BL | 8.16 (8.60) | 0.95 | 88.2 |
| HR (beats/min) |  |  |  |
| Week 4 vs BL | 0.59 (6.63) | 0.09 | 6 |
| Week 8 vs BL | 0.43 (6.33) | 0.07 | 5.6 |
| Week 12 vs BL | 0.94 (7.32) | 0.13 | 7.1 |
| RMSSD (ms) |  |  |  |
| Week 4 vs BL | -2.33 (24.15) | -0.1 | 6.2 |
| Week 8 vs BL | 9.18 (23.46) | 0.39 | 25.5 |
| Week 12 vs BL | 13.51 (31.14) | 0.43 | 30.1 |
| Hypertensive without hypertension medication | | | |
| SBP (mmHg) |  |  |  |
| Week 4 vs BL | 10.40 (9.36) | 1.11 | 77.7 |
| Week 8 vs BL | 10.60 (10.89) | 0.97 | 66.4 |
| Week 12 vs BL | 11.02 (11.05) | 1 | 68.5 |
| DBP (mmHg) |  |  |  |
| Week 4 vs BL | 5.66 (6.97) | 0.81 | 51.2 |
| Week 8 vs BL | 6.02 (8.62) | 0.7 | 40.2 |
| Week 12 vs BL | 6.52 (7.57) | 0.86 | 56.1 |
| MAP (mmHg) |  |  |  |
| Week 4 vs BL | 7.24 (7.45) | 0.97 | 66.3 |
| Week 8 vs BL | 7.55 (8.84) | 0.85 | 55.2 |
| Week 12 vs BL | 8.02 (8.30) | 0.97 | 65.9 |
| HR (beats/min) |  |  |  |
| Week 4 vs BL | 0.86 (8.72) | 0.1 | 5.7 |
| Week 8 vs BL | -0.44 (8.67) | -0.05 | 5.2 |
| Week 12 vs BL | 0.20 (8.73) | 0.02 | 5 |
| RMSSD (ms) |  |  |  |
| Week 4 vs BL | 6.61 (19.21) | 0.34 | 13.5 |
| Week 8 vs BL | 1.71 (24.31) | 0.07 | 5.3 |
| Week 12 vs BL | 22.01 (37.22) | 0.59 | 30.5 |
| Hypertensive with hypertension medication | | | |
| SBP (mmHg) |  |  |  |
| Week 4 vs BL | 13.98 (14.44) | 0.97 | 58.1 |
| Week 8 vs BL | 10.82 (17.09) | 0.63 | 29.4 |
| Week 12 vs BL | 13.17 (13.29) | 0.99 | 60.1 |
| DBP (mmHg) |  |  |  |
| Week 4 vs BL | 5.87 (8.73) | 0.67 | 32.4 |
| Week 8 vs BL | 6.16 (10.63) | 0.58 | 25.3 |
| Week 12 vs BL | 5.91 (8.66) | 0.68 | 33.3 |
| MAP (mmHg) |  |  |  |
| Week 4 vs BL | 8.57 (10.25) | 0.84 | 46.4 |
| Week 8 vs BL | 7.71 (12.48) | 0.62 | 28.2 |
| Week 12 vs BL | 8.33 (9.94) | 0.84 | 46.6 |
| HR (beats/min) |  |  |  |
| Week 4 vs BL | 0.27 (3.83) | 0.07 | 5.3 |
| Week 8 vs BL | 1.48 (1.95) | 0.76 | 39.6 |
| Week 12 vs BL | 1.81 (6.07) | 0.3 | 10.3 |
| RMSSD (ms) |  |  |  |
| Week 4 vs BL | -15.75 (27.08) | -0.58 | 25.5 |
| Week 8 vs BL | 18.16 (21.29) | 0.85 | 47.9 |
| Week 12 vs BL | 0.75 (15.41) | 0.05 | 5.1 |

**Supplementary Table 3.** Post hoc statistical power and effect size estimates for within-group changes in hemodynamic and autonomic parameters across all subgroups at weeks 4, 8, and 12. Δ denotes the unadjusted mean change from baseline (i.e., raw paired differences, not LMM-derived estimates). Cohen’s d effect sizes and corresponding statistical power (1−β, two-tailed, α = 0.05) are reported for systolic blood pressure (SBP), diastolic blood pressure (DBP), mean arterial pressure (MAP), heart rate (HR), and root mean square of successive differences (RMSSD). Results confirm adequate power to detect large BP reductions in hypertensive patients—particularly in the untreated subgroup—whereas HR and HRV outcomes exhibited low power in most groups, reflecting small effect sizes and greater interindividual variability. SD = standard deviation.

|  | **Spearman’s Correlation** | **p-value** |
| --- | --- | --- |
| SBP BL vs ΔSBP |  |  |
| Week 4 | -0.59 | **0.002** |
| Week 8 | -0.33 | 0.113 |
| Week 12 | -0.57 | **0.004** |
| DBP BL vs ΔDBP |  |  |
| Week 4 | -0.60 | **0.002** |
| Week 8 | -0.50 | **0.012** |
| Week 12 | -0.65 | **0.001** |
| MAP BL vs ΔMAP |  |  |
| Week 4 | -0.61 | **0.001** |
| Week 8 | -0.40 | 0.053 |
| Week 12 | -0.64 | 0.001 |
| HR BL vs ΔSHR |  |  |
| Week 4 | -0.16 | 0.458 |
| Week 8 | -0.63 | **0.001** |
| Week 12 | -0.40 | 0.053 |
| RMSSD BL vs ΔRMSSD |  |  |
| Week 4 | -0.56 | **0.006** |
| Week 8 | -0.46 | **0.032** |
| Week 12 | -0.71 | **0.001** |

**Supplementary Table 4** Spearman’s correlation coefficients and p-values for the relationships between baseline systolic blood pressure (SBP), diastolic blood pressure (DBP), mean arterial pressure (MAP), heart rate (HR), and root mean square of successive differences in RR intervals (RMSSD) with their respective relative percentage changes from baseline (BL, week 1) to mid treatment (week 4), end of treatment (week 8) and end of follow up (week 12).

|  | **Spearman’s correlation** | **p-value** |
| --- | --- | --- |
| Voltage vs ΔSBP |  |  |
| Week 4 | -0.03 | 0.900 |
| Week 8 | 0.44 | 0.066 |
| Week 12 | 0.11 | 0.663 |
| Voltage vs ΔDBP |  |  |
| Week 4 | 0.11 | 0.669 |
| Week 8 | 0.22 | 0.385 |
| Week 12 | 0.09 | 0.717 |
| Voltage vs ΔMAP |  |  |
| Week 4 | -0.05 | 0.848 |
| Week 8 | 0.27 | 0.287 |
| Week 12 | 0.11 | 0.651 |
| Voltage vs ΔHR |  |  |
| Week 4 | -0.54 | 0.021 |
| Week 8 | -0.39 | 0.106 |
| Week 12 | -0.31 | 0.203 |
| Voltage vs ΔRMSSD |  |  |
| Week 4 | -0.23 | 0.363 |
| Week 8 | 0.26 | 0.299 |
| Week 12 | -0.01 | 0.971 |

**Supplementary Table 5** Spearman’s correlation coefficients and p-values for the relationships between stimulation voltage and the respective changes in systolic blood pressure (SBP), diastolic blood pressure (DBP), mean arterial pressure (MAP), heart rate (HR), and root mean square of successive differences in RR intervals (RMSSD) from baseline (BL, week 1) to mid treatment (week 4), end of treatment (week 8) and end of follow up (week 12).

|  | **Baseline** | **Week 4** | **Week 8** | **Week 12** |
| --- | --- | --- | --- | --- |
| Non-hypertensive | | | | |
| SBP (mmHg), mean (SD) | 123.9 (10.1) | 122.8 (10.1) | 121.6 (11.4) | 123.8 (10.2) |
| 95% CI | 118.4 to 129.3 | 118.4 to 129.3 | 115.4 to 127.8 | 118.2 to 129.3 |
| DBP (mmHg), mean (SD) | 80.2 (6.5) | 80.6 (5.3) | 79.6 (6.0) | 81.4 (6.0) |
| 95% CI | 76.7 to 83.8 | 77.7 to 83.5 | 76.4 to 82.9 | 77.9 to 85.0 |
| MAP (mmHg) | 94.8 (7.4) | 94.7 (5.8) | 93.6 (7.3) | 95.6 (7.1) |
| 95% CI | 90.8 to 98.8 | 91.5 to 97.9 | 89.6 to 97.6 | 91.7 to 99.4 |
| Heart rate (beats/min), mean (SD) | 75.3 (7.6) | 77.3 (8.7) | 77.4 (7.8) | 80.1 (9.6) |
| 95% CI | 71.1 to 79.4 | 72.6 to 82.0 | 73.2 to 81.7 | 74.8 to 85.3 |
| RMSSD (ms), mean (SD) | 62.0 (19.2) | 61.9 (18.9) | 64.5 (22.6) | 73.5 (76.5) |
| 95% CI | 51.6 to 72.4 | 51.3 to 72.6 | 52.2 to 76.7 | 26.1 to 120.9 |
| Hypertensive | | | | |
| SBP (mmHg), mean (SD) | 151.0 (10.3) | 139.0 (11.9) | 140.3 (15.0) | 139.0 (12.8) |
| 95% CI | 144.9 to 157.1 | 132.0 to 146.0 | 131.5 to 149.2 | 131.5 to 146.6 |
| DBP (mmHg), mean (SD) | 98.3 (3.7) | 92.5 (6.7) | 92.2 (8.2) | 92.1 (6.3) |
| 95% CI | 96.1 to 100.5 | 88.6 to 86.5 | 87.4 to 97.1 | 88.3 to 95.8 |
| MAP (mmHg) | 115.9 (5.1) | 108.0 (8.1) | 108.3 (10.1) | 107.7 (7.7) |
| 95% CI | 112.9 to 118.9 | 103.3 to 112.8 | 102.3 to 114.2 | 103.1 to 112.3 |
| Heart rate (beats/min), mean (SD) | 79.4 (6.9) | 78.8 (7.7) | 79.0 (5.2) | 78.5 (8.6) |
| 95% CI | 75.3 to 83.5 | 74.3 to 83.4 | 75.9 to 82.1 | 73.4 to 83.6 |
| RMSSD (ms), mean (SD) | 63.9 (21.5) | 64.8 (20.9) | 54.7 (23.3) | 51.3 (19.8) |
| 95% CI | 51.2 to 76.6 | 51.8 to 77.7 | 40.9 to 68.5 | 39.0 to 63.5 |

**Supplementary Table 6** Evolvement of blood pressure (BP), heart rate and heart rate variability from study start to end of follow up. Data are presented as mean (standard deviation) for systolic blood pressure (SBP), diastolic blood pressure (DBP), mean arterial pressure (MAP), heart rate and root mean square of successive differences in normal RR-intervals (RMSSD) at baseline (week 1), mid of treatment (week 4), and end of treatment (week 8) and end of follow up (week 12). CI, confidence interval; SD standard deviation. Note: For statistical comparisons, including p-values and effect sizes (Cohen’s d) for within-group changes over time, see **Supplementary Table 3.**

**S1. Subgroup analysis of treated and untreated hypertensive patients**

Untreated hypertensive patients exhibited significant reductions in systolic and mean arterial pressure over time. From baseline to week 12, SBP decreased by 11.0 (11.05) mmHg (p = 0.001), MAP by 8.02 (8.30) mmHg (p = 0.003), and DBP by 6.52 (7.57) mmHg (p = 0.011). These changes corresponded to large within-group effect sizes (Cohen’s d = 1.00, 0.97, and 0.86, respectively), with post hoc power estimates exceeding 65% across all parameters. Heart rate remained unchanged, with a minimal change from 77.5 (8.1) bpm at baseline to 77.3 (8.2) bpm at week 12 (Δ = 0.20 bpm, p = 0.944, d = 0.02). Heart rate variability, measured via RMSSD, declined modestly from 70.3 (26.1) ms to 48.3 (25.5) ms (Δ = −22.0 ms, p = 0.046, d = 0.59), though this change did not remain statistically significant after correction. These results are visualized in Figure 4 and detailed in Supplementary Tables 2 and 5. By contrast, patients receiving pharmacological treatment also showed reductions in BP (e.g., SBP: −13.2 mmHg at week 12), but these did not reach statistical significance (all p > 0.05), likely due to higher baseline variability or medication-related confounding. HR and RMSSD remained stable across time points. Taken together, these findings suggest that aVNS led to clinically meaningful and statistically significant BP reductions in untreated patients, with effects persisting into follow-up. In contrast, BP reductions in treated hypertensives were more variable and did not achieve statistical significance. HR and HRV remained largely unchanged across all groups, indicating that the BP-lowering effects of aVNS occurred without broader alterations in autonomic tone. Supplementary Figure 2 illustrates individual trajectories in BP and HR, highlighting interindividual variability across subgroups.

In the subgroup of untreated hypertensive patients, stimulation voltage was significantly associated with reductions in SBP (β = –0.02 mmHg/mV, p < 0.001) and MAP (β = –0.01 mmHg/mV, p < 0.001), indicating a dose–response effect of stimulation intensity. Similarly, age and BMI emerged as significant covariates in some subgroup models. For instance, BMI was positively associated with SBP in the treated hypertensive subgroup (p = 0.034), while age showed a modest positive association with DBP in non-hypertensive patients (p = 0.045). These findings suggest that demographic factors may modulate the response to aVNS and merit further investigation in larger, powered studies. Full fixed-effect estimates for all subgroup-level models are provided in Supplementary Table 4.


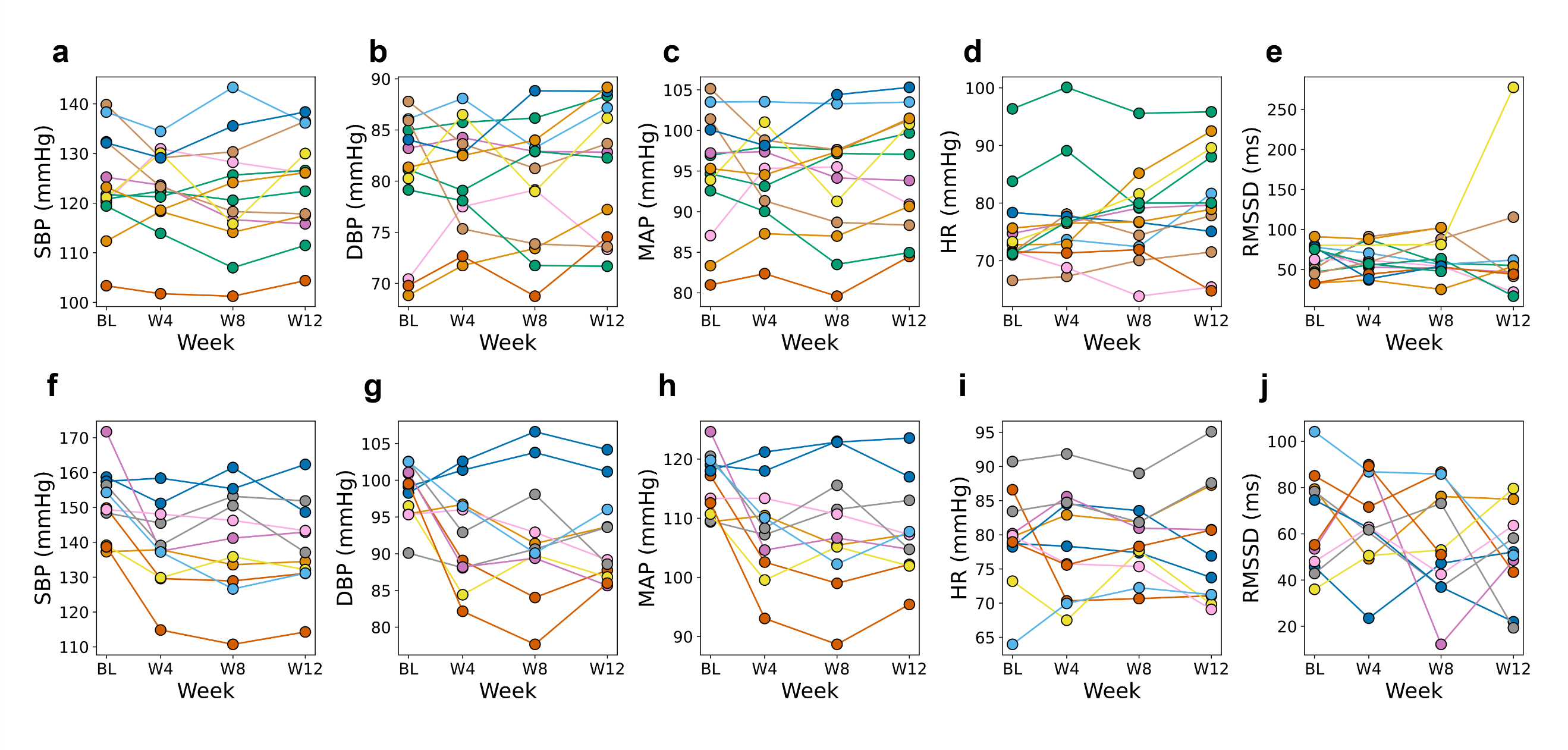


**Supplementary Figure 1** Individual trajectories of systolic blood pressure (SBP), diastolic blood pressure (DBP), mean arterial pressure (MAP), heart rate (HR), and root mean square of successive differences (RMSSD) across baseline (BL, week 1), mid of treatment (week 4), end of treatment (week 8), and end of follow up (week 12) in non-hypertensive (**a-e**) and blood pressure (BP) reductions were observed in hypertensive chronic pain patients undergoing auricular vagus nerve stimulation (aVNS). (**f-j**) patients. Each colored line represents an individual subject, illustrating variability in responses to aVNS over time.


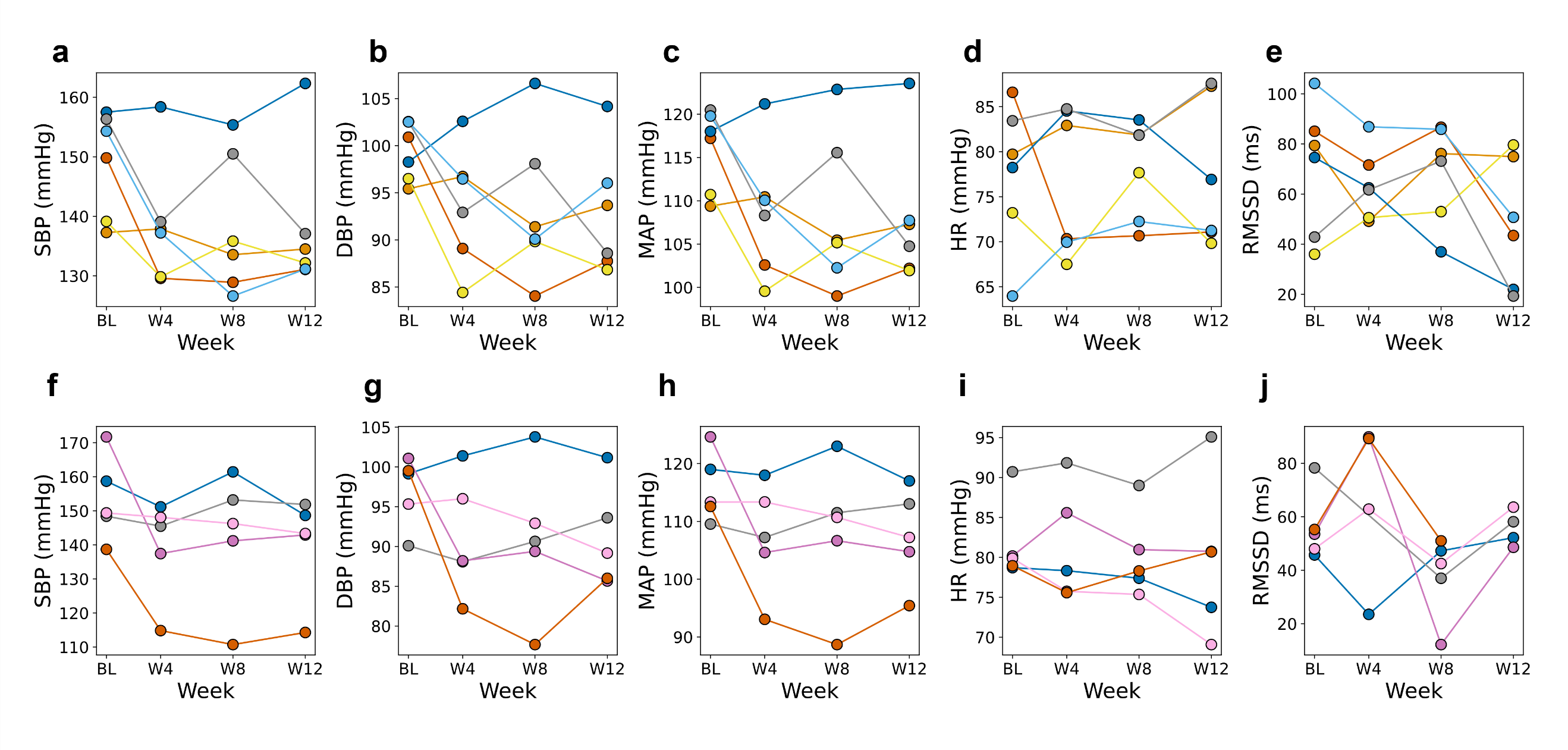


**Supplementary Figure 2** Individual trajectories of systolic blood pressure (SBP), diastolic blood pressure (DBP), mean arterial pressure (MAP), heart rate (HR), and root mean square of successive differences in RR intervals (RMSSD) across baseline (BL, week 1), mid of treatment (week 4), end of treatment (week 8), and end of follow up (week 12) in hypertensive patients without hypertension medication (**a-e**) and hypertensive patients with hypertension medication (**f-j**) Each colored line represents an individual subject, illustrating variability in responses to aVNS over time.
